# Improving the use of focus group discussions in low income settings

**DOI:** 10.1186/s12874-020-01168-8

**Published:** 2020-11-30

**Authors:** Pauline F. D. Scheelbeek, Yashua A. Hamza, Joanna Schellenberg, Zelee Hill

**Affiliations:** 1 Department of Population Health, London School of Hygiene & Tropical Medicine, London, UK; 2 Childcare & Wellness Clinics, Abuja, Nigeria; 3 Department of Disease Control, London School of Hygiene & Tropical Medicine, London, UK; 4 University College London, Institute for Global Health, London, UK

**Keywords:** Focus group discussions, Bias, Qualitative data collection, Low-income settings, Activity-oriented exercises

## Abstract

**Background:**

The quality of data obtained through Focus Group Discussions (FGDs) is highly dependent on appropriate design and facilitation. In low-income settings steep power gradients between researcher and participants, as well as conversational norms, could reduce the ability of participants to voice personal opinions. Activity-oriented exercises have been suggested as a way overcoming these challenges, however little evidence exists - to date - on their use in low-income settings. We selected six exercises for use in Ethiopia and Nigeria and report our experiences.

**Methods:**

The six exercises (picture sorting, associative pictures, picture ranking, decision trees, predictive story-telling and provocative statements) were used in 32 maternal and new-born care themed FGDs conducted in Amhara and Southern Nations Nationalities and People’s Regions (Ethiopia) and Gombe State (Nigeria). Six facilitators and two supervisors who used these exercises were interviewed about their experiences. FGD verbatim transcripts and interview notes were analysed to explore methodological effectiveness and respondents’ experience. All data were coded in NVIVO using a deductive coding frame.

**Results:**

Facilitators and participants described the methods as ‘fun’ and ‘enjoyable’. The exercises yielded more in-depth and complete information than ‘normal’ FGDs, but facilitator’s probing skills and overall FGD group dynamics proved crucial in this success. Explaining and conducting the exercises increased FGD length. Data richness, participant reaction and understanding, and ease of facilitation varied by study site, exercise, and participant group. Overall, the exercises worked better in Nigeria than in Ethiopia. The provocative statement exercise was most difficult for participants to understand, the decision-tree most difficult to facilitate and the picture exercises most enjoyable. The story telling exercise took relatively little time, was well understood, yielded rich data and reduced social desirability bias.

**Discussion:**

The majority of the exercises proved successful tools in yielding richer and less biased information from FGDs and were experienced as fun and engaging. Tailoring of the exercises, as well as thorough training and selection of the facilitators, were pivotal in this success. The difference in the two countries shows that adequate piloting and adaptation is crucial, and that some exercises may not be adaptable to all settings.

**Supplementary Information:**

The online version contains supplementary material available at 10.1186/s12874-020-01168-8.

## Background

Focus group discussions (FGDs) have been used in public health research since the 1990s [1]. They aim to explore participants’ experiences, beliefs, and attitudes by using group processes to stimulate responses and gain insights through participants exchanging views, and questioning and challenging each other [1–5]. They are often perceived as a cost and time efficient way of collecting information from multiple participants; and it is this efficiency, rather than their methodological strengths, that is often highlighted in the literature [2]. FGDs are commonly conducted as group interviews (hence losing the advantages that an FGD bring to data collection), with interaction between the facilitator and each participant in turn, rather than between participants [6, 7]. As a result, the quality of the research they produce has been questioned [1, 7].

Most of the literature on how to conduct FGDs comes from high income settings [7, 8], and guidelines for low income settings have been criticized for focusing on practicalities, rather than on managing contextual challenges in the interactive process [9]. Challenges include steep power gradients between researcher and participants, and conversational norms, reducing the ability of participants to voice disagreements, or provide personal opinions [7]. We have used focus groups in Ghana for over 15 years, and have had difficulty making FGDs interactive, with both facilitators and participants seemingly more comfortable with group interviews. Over this time we noticed that the level of participant-participant interaction increased during activity oriented exercise, a similar experience reported by researchers in Tanzania [7].

Based on a review of the literature and our experiences in Ghana, we decided to trial the use of a variety of exercise. As well as encouraging interaction, we wanted to see if using exercises could improve the quality of our data around the drivers of behavior change. Understanding behaviors can be challenging as individuals can have difficulty explaining their own behaviors, may be unaware of some of the factors that influence their behavior, or there may be social desirability bias in reporting (e.g. [10, 11]). These issues can lead to participants providing an unrealistic impression of their behaviors, giving superficial answers and being strongly influenced by probes [12–14]. Exercises may help reduce these problems by encouraging respondents to reflect on their behavior and by provoking gut reactions that uncover hidden reasons for behaviors and reduce social desirability bias. They can also focus participants on what ‘other’ people do, this may improve data quality as people are often better at explaining the behavior of others rather than their own, and because projecting their answers onto others may reduce social desirability bias [15, 16].

Activity oriented exercise, such as sorting, story-telling and sentence completion, have been suggested as a way of improving focus groups for over two decades [6]. They are described as a means to make a group more enjoyable, increase interaction, reduce boredom, focus attention, increase reflexivity, and make sensitive topics less threatening [5, 6]. Despite their potential advantage social scientists often rely on discussion questions, whilst exercises are more widely used in market research [9]. We found little written about the use of these exercises in low- income settings.

During the planning for a study in Ethiopia and Nigeria, we conducted a scoping literature review to identify exercises that could enhance our data collection. We searched PubMed/MEDLINE, Web of Science, Scopus, and Cochrane, screened relevant websites and expert forums for grey literature, conducted internet searches, and asked experts for relevant citations. We included literature from fields such as public health, sociology, market research, economics and criminology. We identified 36 exercises and generated a set of tables where we described each exercise, reflected on how it could be used in the study, on its advantages and disadvantages and on which biases it may help overcome. Based on these reflections we then scored each method as having high, medium or low potential for overcoming social desirability bias, improve enjoyment and yield rich in-depth data for each of the study behaviours in our study settings. These tables can be found in the supplementary files. We then met as a study team to review the tables and selected six exercises for use. This paper describes our experiences using these six methods.

## Methods

The six exercises were used as part of a study on maternal and newborn care practices that explored the role that community health workers play in behaviour change [17–19]. The research, in Ethiopia and Nigeria, consisted of 49 interviews with recent mothers, 13 “friendship pair” interviews – in which mothers and a friend were interviewed together – and 32 FGDs with mothers, grandmothers, fathers and community health workers.

In Ethiopia, we conducted the study in four *Woreda* (districts) in Amhara and The Southern Nations, Nationalities and Peoples (SNNP) Regions. The *Woreda* were selected because they had relatively well functioning community health workers and were ‘typical’ – that is they had no unusual characteristics in terms of employment, accessibility, or population. Within each *Woreda* we selected a *Kebele* (smallest administrative division) that was reasonably accessible to the data collection team. The majority of respondents worked in agriculture and were typical for the area in terms of socio-economic class and education. The age of mothers in the FGDs ranged from 18 to 30 years old (mean = 26) and the mean reported number of children was 3.1 (range 1–7); for fathers and grandmothers age ranges were 28–45 years (mean = 36) and 36–75 years (mean = 55), with a mean reported number of children of 4 and 5 respectively. The median number of years in education was 3 for mothers and 5 for fathers, and of all interviewees 69% were Christian (predominantly Orthodox) and 31% Muslim. The interviewed community health workers had an average of 8.5 (Health Extension Workers - HEWs) and 1.9 (Health Development Army volunteers- HDAs) years of experience.

In Nigeria, we collected data from two Local Government Areas (LGAs) in Gombe State, a heterogenous state comprising multi-ethnic groups of which Fulani is the largest. One selected LGA was predominantly Christian and the other Muslim. Within each LGA, two communities were selected: the LGA headquarters and an accessible rural community that, for security reasons, would allow the researchers to be back in Gombe City before sunset. The age range of mothers in the FGDs ranged from 20 to 40 years of age (mean = 28) and the mean reported number of children was 3.9 (range 1–9); for fathers and grandmothers age ranges were 30–61 years (mean = 43) and 30–70 years (mean = 53), with a mean reported number of children of 7.7 and 5.8 respectively. Interviewees in Nigeria had usually received more years of education than those in Ethiopia: the median number of years in education was 6 for mothers and 12 for fathers. Over a third (37%) of all interviewees were Christians, whilst all others were Muslims. The interviewed frontline workers had an average of 2 (Federation of Muslim Women’s Associations in Nigeria Volunteers– FOMWANs) and 3.2 (Traditional Birth Attendants – TBAs) years of experience.

The FGD data were collected in 2015, by four interviewers in Ethiopia and six in Nigeria. The Ethiopian interviewers, 2 male and 2 female, had on average 5 years of experience in qualitative data collection and were skilled in facilitating focus group discussions. They were native speakers in the languages in which FGDs were conducted, apart from Silte, where we used translators. Cross-religious interviewing did not pose a problem, but focus groups with fathers were all conducted by male interviewers. The Nigerian interviewers, 3 male and 3 female, had varying experience in qualitative data collection (between 2 and 17 years), and were native speakers of the languages used in the FGDs. The more junior interviewers took the role of note takers, whilst the more senior interviewers facilitated the groups. Religion of facilitators and participants were matched, but interviewers facilitated FGDs with both sexes.

Facilitators and translators had 4 days of classroom training, including 2 days focusing on the activity-oriented exercises. They had 2 days of training on verbatim transcript writing, administration and data safety & confidentiality issues. During the training we explained the objectives of each exercise, reviewed the discussion guides and conducted role plays. This was followed by a two-day pilot test, where the semi-structured guides were adjusted, the exercises discussed and problems resolved. The FGDs consisted of 3–7 participants and were conducted in neutral locations such as community centres, the length of the FGDs are shown in Table 1. Study aims were explained and written consent was obtained from each participant (or their designated proxy if the participant was illiterate) before the start of the FGDs. The development of the FGD topic guides has been described in detail elsewhere [17–19].

**Table 1:** Exercise used by participant group

Participant group	Exercise used	Mean length Ethiopia	Mean length Nigeria
Mothers	Picture pile sorting Decision tree Predictive story telling Provocative statements	2 h	1 h and 15 mins
Grandmothers	Associative pictures Picture ranking Predictive story telling Provocative statements	2 h 40 mins	1 h and 15 mins
Fathers	Associative pictures Predictive story telling Provocative statements	1 h and 40 mins	1 h and 20 mins
CHWs	Picture pile sorting Decision tree Provocative statements	1 h and 40 mins	1 h and 10 mins

All FGDs were audio-recorded and transcribed by the interviewers in English within a week of data collection. During data collection, interviewers received regular feedback from senior researchers on their facilitation techniques and the use of the exercises. We also held a review meeting in the middle of data collection, to reflect on our findings and experiences and adjust data collection as needed.

Each exercise is described below along with their aims. Increasing interaction between participants was an aim of all the exercises and is not listed. The exercises aimed to be culturally acceptable, salient to participants and feasible to conduct. This included adjusting exercises where literacy was required.

### Picture pile sorting

Participants were asked to work together to put seven pictures of newborn care practices into piles of those commonly practiced or not commonly practiced in their community. They were then asked to pile the same pictures into practices they felt were important or not important for the health of the baby. Finally, they were asked to pile the pictures into practices that that were promoted, or not promoted, by Community Health Workers (CHW). Facilitators probed on reasons for the classification and encouraged participants to provide details of the practices. The exercise aimed to focus the participants on the task rather than on the facilitator and increase reflexivity. Pictures for this exercise (as well as the associative picture and picture ranking exercises) were carefully selected with the help of facilitators, local government officials and community health workers. Where needed, pictures were taken in the local area to ensure they reflected the local context.

### Associative pictures

Participants were shown a picture of a newborn care practice and asked to give their immediate reaction to the picture. Facilitators probed to understand the reaction. In total 5 pictures were shown. Their responses were used to facilitate further discussions within the group. By encouraging an immediate reaction, we aimed to capture the participants’ first emotions and responses to the pictures, with the aim of decreasing social desirability bias in their answers and uncovering hidden drivers of behaviour.

### Picture ranking

Participants were shown pictures representing the immediate family, a neighbour and a CHW. They were asked to rank the people from most to least influential on newborn care practices. If two or more people had equal influence they could be placed next to each other. After ranking the pictures, the participants were asked about any ranking differences for specific care elements, such as bathing, feeding and thermal care. This exercise aimed to focus the participants on the task and increase reflexivity.

### Decision tree (root-cause analysis)

A trunk of a tree, its main roots and sub roots were drawn on a large flipchart. The central problem was written down on the trunk of the tree. Participants were then asked to give underlying reasons for why the problem may occur. The sub roots were subsequently used to document “reasons for those reasons”. When applicable further subdivisions were made to determine the core of the problem. The exercise aimed to yield in-depth answers for complex questions by triggering the participants’ cognitive skills and encouraging them to think in a more comprehensive and reflexive way about the problem presented.

### Predictive story telling

The facilitator read a fictitious, but realistic, story that needed to be finished by the participants. The story explored a dilemma new mothers may face when receiving conflicting advice from family members and community health workers. Once the first part of the story was completed, participants were given some new information and asked to say what would happen next. The exercise aimed to reduce embarrassment and social desirability bias, as participants can give their answers in the third person (i.e. as if they are talking about the fictitious person) whilst reflecting on their own practices.

### Provocative statements (Q-statements)

A series of controversial statements were read out to the participants, who were encouraged to respond as soon as the facilitator finished the statement. As for the associative picture exercise, this exercise aimed to capture gut reactions, to reduce social desirability bias and uncover hidden drivers of behaviours.

The exercises described above were used in FGDs with mothers, grandmothers, fathers and community health workers. Each respondent group had a specially designed interview guide that included 3–4 of the selected methods as shown in Table 1.

Our experiences with the exercises were evaluated in three ways: Interviews with and reflections of senior researchers who observed 16 of the 40 pilot and actual FGDs, and who supervised the data collectors; skype or phone interviews with eight of the 10 data collectors about their experiences and views on the exercises; and a review of all the FGD transcripts to explore how well the methods worked and how the respondents reacted to them. The skype or phone interviews were conducted in 2015, 4 months after the completion of data collection. Interviews were conducted in English by one of us (PS) using a semi structured guide which asked about overall impression of the exercises in the FGDs, advantages and disadvantages of each exercise, as well the ability of the exercise to reach its objective, such as yielding rich and valid data, overcoming social desirability bias, participant engagement, and enjoyment of the exercises for participants and facilitator. Furthermore, facilitators were asked whether they had any recommendations for improvement for each of the exercises. (Supplementary Files) Notes were taken during the interviews, which were written up as expanded notes immediately after the interview.

Data analysis was conducted by PS and ZH who met regularly during the analysis process to discuss the coding and emerging themes. Analysis started with multiple readings of the expanded notes and FGD transcripts to ensure familiarity. Analysis was conducted in Nvivo with nodes for each exercise, and was both deductive and inductive. The deductive themes were: data quality (sub themes were data depth/richness, interactions between participants and impact on social desirability bias); feasibility (sub themes were ease of facilitation, participant comprehension and impact on FGD length) and participant reaction (sub themes enjoyment).

Analysis of the FGD transcripts included coding all participant comments on the methods (e.g. we are enjoying this, I don’t understand this, this is taking too long) and any other reactions (e.g. laughter, silence, confusion, enthusiasm, distraction). For the sub-theme data depth/richness we coded examples of the length and depth of responses and for impact on social desirability bias examples of guarded responses, frank/open responses and any statement where respondents were critical or the services they had received. To put these findings in context we generated memos for each FGD on the overall group dynamics, the facilitators skills in probing and managing the group and anything that seemed to make a method work well or nor so well.

The skype or phone interviews and the FGDs were analyzed separately and the main themes and findings were then compared. This was bi-directional as we explored whether the findings from the interviews with study staff were reflected in the FGD data (e.g. if the staff said a method was challenging for participants could we see evidence of this in the FGD transcripts) as well as exploring if the study staff interviews could explain the results of the FGD analysis (e.g. if a method garnered very rich FGD data could the staff interviews help understand why?). This resulted in a set of final themes that reflected both the interview and the FGD transcript data.

## Results

The final coding tree was similar to the initial deductive themes that are described above. Inductive sub-themes emerged under ‘feasibility’ relating to issues of data processing (e.g. note taking, transcription and translation) and the constraints of physical space; and for ‘participant reaction’ relating to distraction and emotional responses. Below we present our overarching findings on the exercises followed by exercise-specific findings.

The facilitators felt that the exercises yielded more in-depth and complete information than the *‘normal’* FGDs they were used to. This was echoed by the principal investigator (ZH) who found the FGDs more discursive, and less of a group interview, than previous FGDs in the study settings. Both facilitators and participants describe the methods as ‘*fun’* and ‘*enjoyable’*:

*‘Some of the participants we selected for the in-depth interviews* [with no “exercises”] *came out very disappointed: they heard from friends that it was something fun with pictures and drawings, but in their own interview only “normal” questions were asked’* [FGD facilitator- Nigeria]*.*

Data richness, participant reaction and ease of facilitation varied by study site, exercise, and to a lesser extent participant group. In relation to study site, overall the methods worked better in Nigeria than in Ethiopia, as they were better understood. The facilitators felt that participants were more open and interacted with each other more than in Ethiopia. This was also true for the non-exercise elements of the FGDs.

In both sites there was evidence of social desirability bias, which varied by FGD. Some FGDs resulted in frank and illuminating discussions and others in guarded and short responses – unsurprisingly the exercises worked best in the more open FGDs. Facilitators put the variation down to group dynamics, for example, the presence of a domineering and judgemental participant in one FGD appeared to stop participants opening up, while in another FGD a very open participant shared a personal story which prompted others in the group to be more open. As well as group dynamics, the depth of the data was linked to the ability of the facilitator to probe effectively without turning the FGD into a group interview.

The number of exercises used in the FGDs was relatively high (3–4), and took longer than we had anticipated, particularly in Ethiopia. Some facilitators felt that this was too many, and caused exhaustion towards the end of the FGDs for both participants and facilitators. We had many examples of participants having to leave, asking when the FGD would end and being distracted.

Respondent 2: Ar*e we not going?*

Facilitator: *We are only left with two questions.*

Respondent 2: *In Christ’s sake*

Respondent 3: *It is getting late* [Mother FGD- Ethiopia]

Using the same methods across respondent groups, allowed us to compare and contrast findings.

On a practical level the note-takers and transcribers experienced difficulties in capturing the discussion when the exercises yielded excitement or heated discussion as multiple participants often talked at the same time or in quick succession. Where translators were used valuable discussions that occurred while exercises were being conducted were lost.

Our findings for each method are presented below and are summarized in Table 2.

**Table 2:**
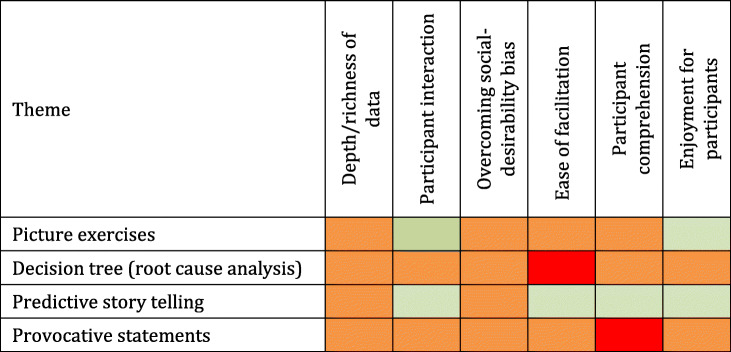
Identified themes and positive, mixed and negative experiences in exercises used by participant group

_*=Predominantly positive experiences;*_ _*= Mixed experiences;*_ _*= Predominantly negative experiences*_*.*

### Associative picture, ranking & sorting exercises

The pictures were often greeted by participants with enthusiasm, and sometimes an emotional response: *‘poor woman …*. ’ or *‘it* [picture] *gives me joy’*; and participants enjoyed the exercises:

Facilitator: *Okay, thank you, we will revisit some of these discussions later, but let me gather these pictures.*

Respondents: *All clap.*

Respondent 1: *We are enjoying this, we like this discussion with the pictures* [clapping continues] [Grandmother FGD Nigeria].

Despite this enthusiasm the exercises were not always easily understood, and facilitators sometimes explained the exercise multiple times, had to reorient discussions, or had to sort or rank the pictures themselves using multiple prompts *‘who is the next influential … … and then who’*. This added to the length of the FGDs. Although there were some comprehension problems in Nigeria: *‘Can you explain it to us again’,* these were significantly less than in Ethiopia. This is reflected in a greater facilitator- participant interaction during the exercises in Ethiopia, compared to greater participant-participant interaction in Nigeria.

The facilitators themselves described the exercises as *‘hit and miss’*, particularly the associative picture exercise, but felt they were a useful starting off exercise:

‘*Excitement in this round did not always lead to useful discussions [ … ] and quality of answers, but gave an initial overview. This is a great exercise at the beginning, as it could serve as some sort of ice-breaker. Or it could come back at several points during the interview to re-energise the participants’* [Facilitator Nigeria].

Overall these exercises yielded useful information about behaviours that were practiced, beliefs about these behaviours, advice given about the behaviours and key influencers. The level of discussion was linked to whether the topic of the picture was of a new or unusual behaviour, or whether there were variations in opinions about the practice of the behaviour. For example, the first quote below is for wrapping the baby after delivery, this is a widely accepted behaviour with little variation in beliefs. The second quote relates to skin to skin care, a new practice, where discussions of the topic were livelier and in depth. A lack of discussion did not mean the exercise did not work well, as it told us something about the normality of the behaviour:

Facilitator: *Let me show you another picture. It shows a baby being wrapped after delivery. [ … ] What do you think about this?*

Participant 1: *It is good*

Participant 2: *It is good*

All respondents (mothers) upon seeing a skin-to-skin picture: *It is not practised! [laughter]*

Respondent 1: *Are they naked?*

Facilitator: *Yes.*

Respondent 1: [laughs]

Respondent 2: [looks surprised by the picture]

Respondent 1: *We don’t see such thing before … ...*

It was essential that the facilitator explained the picture before starting the exercise. Otherwise discussions deviated into the details of the picture, such as whether the person was from their ethnic group, was the baby circumcised, or that the mother looked tired. Another problem was our initial use of too many, and too similar, sorting and ranking exercises. This led to repetition in content, which frustrated the participants:

‘Respondent 1: *We have already discussed on it. You are asking repeatedly.*

Facilitator: *It is not a repeated question. Earlier we have discussed on the most important and less important behaviours. Now I am asking you about what the Health workers promote and encourage you to do and do not’* [Mother FGD- Ethiopia].

Other issues that came up less frequently were participants believing that we were looking for the correct answer rather than an opinion; a lack of space making the sorting and ranking difficult to do; and poor eyesight in the older respondents meaning that pictures in the associative picture exercise needed to be passed around rather than held up, which added substantially to the time the FGD took.

### Decision tree (root-cause analysis)

The root cause analysis was described by all facilitators as useful, but challenging. Overall, they provided rich information on the problems they explored, especially in the FGDs where the participants were open and felt able to be critical. In these cases, participants were eager to share their opinions and knowledge:

‘Facilitator: *What are the reasons why women do not go and deliver at the health facility as desired?*

Respondent 1: *Here I am* [raises hand eagerly]

Respondent 2: *Here I am too, I have much to say* [raises hand].

Facilitator: “*Good! Let us get the main reasons’* [community health workers FGD- Nigeria].

The depth of data was also linked to whether the participants perceived that the chosen topic was a problem – probably linked to their openness. For example, in one FGD participants repeatedly said there was no problem and all was being done well, yet in a second FGD participants shared their personal experiences of the problem, which provided insightful and rich data.

Not all participants understood the symbolic meaning of the tree trunk and the roots, and from the transcripts it appears that the drawings were not always used. Facilitators reported that writing the reasons for the problem on the tree root helped to structure the participants’ thoughts and deepen out emerging issue. Writing down the participants’ reasons was difficult for illiterate participants, and the facilitators tried to overcome this by reading the labels out loud and summarizing them every now and then.

Facilitators found it challenging to process all information to make an adequate and informative root diagram on the spot, especially as participants often gave answers in an unstructured manner. The exercise was regarded as the most demanding for facilitators, requiring a high level of analytical skills. Furthermore, some facilitators suggested that the tool worked better with more educated participants, such as the community health workers, who themselves had better analytic skills.

### Predictive storytelling

Participants identified with the character and the situation in the story, and answered questions as if the character was living in their village. Some facilitators opened the story as if telling a folk tale which participants liked. With the help of facilitator probes the scenarios generated discussions and uncovered insights such as how families manage conflicting advice, and the level of trust in community health workers: *“We were able to get interesting information on mechanisms of change. There were arguments for both sides of the story and they explored several scenarios”* [Facilitator- Ethiopia]. The method was relatively quick to do in relation to the quantity of data captured.

Facilitators felt that focusing on a character reduced social desirability bias. For example, participants said the character may behave at odds with expected behavior, but then stressed that they themselves would behave differently: *‘She will listen to her sister’s advice, but as for me it is better if she follows the health worker advice’* [Mother FGD Ethiopia]. In most FGDs participants switched between the third and first person when talking about the scenarios, with the first person used to present a strong belief about a behavior, or to reflect on what they would do differently to the character: *‘NO … NO … NO … …*. *I do not want to do it like this’* [Grandmother FGD Ethiopia].

In Ethiopia participants frequently reported what the character *‘should’* do, rather than on what they ‘*would’* do. Answers tended to be shorter and less complex than in Nigeria, where discussions were often lively, humorous and more open:

‘Respondent 3: *Her mother has right over her … she will follow the mother’s advice, she will be afraid of God’s wrath*

Respondent 2: *but some Ladis* [character] *will not behave like this, the Ladi of nowadays* [laughter], *the rude Ladis of nowadays, who will look into our eyeballs and tell us that the doctors have told them this and that* [imitating Ladi and shaking her body – everyone laughs]

Respondent 1: *They will not keep quiet and listen’* [Grandmother FGD Nigeria]

The switching between the first and the third persona, and between should and would, was an entry point for facilitator probes.

### Provocative statements

The use of statements was often poorly understood by participants, but the exercise did work once participants understood the task. The exercise elicited some useful information, for example it uncovered differences in perceptions of volunteer workers compared to paid community workers, recent changes in who influences behaviors and it allowed us to gauge the strength of people’s opinions. The method did not always yield immediate gut reactions, but there were some successes- particularly in Nigeria: *‘People literally jumped up to give their answers – I had the feeling they really told me their gut feeling, the first thing that came to mind’ [*Facilitator- Nigeria].

Provocative statements worked best where there was a clear understanding that this was a statement to discuss, and not a fact or the facilitators view. It was the method that participants asked the facilitator to explain again most often, and where participants sometimes remained in silence: *‘This topic is difficult for us, please clarify’* [Community health worker FGD, Nigeria]. This occurred despite facilitators conducting practice rounds with humorous examples. The lack of understanding was attributed to confusion between a statement and someone’s opinion: *‘They see an “authoritative” figure and confuse the statement for their opinion. Sometimes they even said, “Is that really true?” whereas it was meant to be provocative’* [Facilitator- Ethiopia]. Faced with silence or confusion the facilitators had to explain the exercise again or rephrase the statement as a *‘normal’* question: *‘On many occasions, we had to convert Q-statements to questions. I think it is more a cultural thing: people are just not used to being confronted by provocative questions. It is either you are asking them a question, or you are telling them something’* [Facilitator-Ethiopia]*.*

Short and focused provocative statements were most suitable in both sites as they were easier to understand. We encountered some problems with translation and with the use of translators. The provocative statements were originally written in English and translated in Amharic and Hausa. For most statements, this did not pose any difficulties, but some statements were difficult to translate. This resulted in long descriptive read outs that were not suitable for use. In these cases, we developed alternative statements written directly in the local language. Where we used translators during the FGD we trained them around the use of provocative statements, but we still encountered problems: *‘The translator had difficulties in interpreting the statements properly, resulting in very long statements, that did not provoke any immediate response’* [Facilitator- Ethiopia].

The provocative statements were done at the end of the FGDs, and the depth and usefulness of responses was linked to how lively the participants were at the point, and whether they perceived that they had provided similar answers previously.

## Discussion

There was agreement across fieldworkers, supervisors and senior investigators that the exercises improved interaction between participants, enhanced data quality, and made the FGDs more enjoyable compared to previous experiences in the settings. We did not encounter a limitation identified in the literature of participants not liking the idea of playing ‘games’ or thinking the researchers were strange [5, 6].

The methods were more successful in Nigeria than Ethiopia: this may be related to cultural differences in conversational norms and issues of positionality between facilitator and participants, exemplified by an Ethiopian facilitator describing that participants saw them as an ‘*authoritative figure’*. The potential for high levels of social desirability bias has been noted for Ethiopia given the political and cultural context [20], and although we found evidence that social desirability bias remained we found that some techniques reduced it, with the story telling approach for example allowing a projection of responses onto an ‘other’. In Ethiopia in-depth ethnographic methods could be a more useful tool to overcoming such bias than FGDs.

A strong influence of positionality on participants was also found in Tanzania, where exercises reduced its impact as participants focused less on the facilitator, with the best FGDs occurring when the facilitator removed themselves from the process altogether [7]. Given the importance of probes for generating rich data in our study, and in other studies in similar settings [21], we would not recommend the removal of the facilitator, but our findings highlight the importance of the selection and training of data collectors, and in-depth thinking about how they present themselves to the participants. We found that key to success of the exercises was, whether the facilitators had fully understood the aims and objectives of the discussions, mastered the content and process of the exercises and knew how to probe effectively. Some of the Nigerian FGDs were facilitated by one of the senior researchers (YH), and the impact on data quality of their greater experience in encouraging interaction between participants and their gentle but effective probing was clearly visible.

The predictive story telling was one of the most successful methods it was well understood, enjoyed by participants and facilitator, generated interaction and its projective nature seems to have gone some way to address social desirability bias. We found one study that reported on the use of stories in a low-income setting, albeit in in-depth interviews [21]. They also concluded that the method had been successful and increased the quality of their data, but faced the same challenge of respondents often reporting what should be done rather than what the character in the story would have done. We discovered that in some languages the differences between should and would were also difficult to translate. We think that one of the reasons the story was successful was because it was carefully tailored to the cultural context and resonated with the participants.

The provocative statements worked the least well with comprehension issues in both sites- despite facilitators using humorous examples. This meant that facilitators spent time explaining the exercise, lengthening the FGDs, and in Ethiopia facilitators often had to rephrase the statements as questions. We also found issues of translation and of using translators particularly problematic for this method, with statements written directly in the local language working best. Language issues are a common problem in FGDs in low income settings with the use of translators having a negative impact on group interaction and discussion, and where at all possible they should not be used [8].

We felt that the number of methods included in the FGDs and repetition of content hindered their effectiveness by making the FGDs too long and at times frustrating for the participant. Although the exercises were described as fun, towards the end of the FGDs participants were often anxious to leave. In Ethiopia the need to explain the exercises multiple times and the use of translators meant the FGDs were longer than we would have hoped. There is no recommendation on the number of exercises that should be included in FGDs, but the numbers should be influenced by the impact they have on the length of the FGDs, participants’ age and ability to express themselves [6].

This is – to our knowledge – the first study reporting on experience with a range of activity-oriented exercises in low-income settings. The study was however subject to some limitations. First, data collection focused on maternal and child care behaviors and the findings may not be transferable to studies accessing other types of behaviors. Second, participants were not asked directly about their opinion and experiences of participating in the FGDs, rather conclusions were drawn from transcripts. Third, the time and scope of the research did not allow for exploration of other methods and exercises beyond the six evaluated in this study: there are several other promising methods that should be explored for use in low-income settings. Finally, interviews with facilitators occurred by phone and skype, after completion of the FGD work in both countries and were conducted by a researcher who had supervised data collection rather than someone external to the study team.

## Conclusions

The majority of our exercises– and in particular story-telling - proved successful tools in yielding rich and less biased information from FGDs. Participants and facilitators experienced the exercises as fun: they improved participation and engagement. However, the exercises adversely affected the length of the FGDs and in future we would use fewer exercises. Given the differences we noted between the two study sites, we recommend that all exercises are adequately piloted and adapted, or not used at all if they do not improve quality.

## Supplementary Information

**Additional file 1.**

**Additional file 2.**

**Additional file 3.**

**Additional file 4.**

**Additional file 5.**

**Additional file 6.**

**Additional file 7.**

**Additional file 8.**

**Additional file 9.**

**Additional file 10.**

## Data Availability

The datasets generated during and/or analyzed during the current study are not publicly available due to issues of confidentiality and privacy. Although respondents’ names are not included in transcripts they do include place names and any names mentioned by respondents, for example, names of Community Health Workers. Restricted and summarized data are available from the corresponding author on reasonable request.

## Abbreviations

FGD: Focus Group Discussion
SNNP: Southern Nations, Nationalities and Peoples
HEW: Health Extension Worker
HDA: Health Development Army
LGA: Local Government Area
FOMWAN: Federation of Muslim Women’s Associations in Nigeria Volunteer TBA Traditional Birth Attendant
CHW: Community Health Worker
