# Supplementary material for: Improving the use of focus group discussions in low income settings
Source: BMC Med Res Methodol. 2020 Nov 30;20:287. doi: 10.1186/s12874-020-01168-8 (PMC7706206; doi:10.1186/s12874-020-01168-8)
Supplement: Supplementary file 1 — Additional file 1. [file 12874_2020_1168_MOESM1_ESM.docx]

**Focus Group Discussion Fathers**

# Theme 1: Socio-demographic and interview information

| - 1. FGD ID:   2. Interview date:   3. Interview start time:   4. Interview end time: | - 1. Interviewer code:   2. Note taker code:   3. Translator code:   4. Tape recording number: |
| --- | --- |

| **Respondent number** | **Age** | **Last completed grade** | **Occupation** | **Ethnicity & religion** | **No. children** | **Place of delivery of last child** | **Age of last child** |
| --- | --- | --- | --- | --- | --- | --- | --- |
|  |  |  |  |  |  |  |  |
|  |  |  |  |  |  |  |  |

# Theme 2: Attitudes and response to the behaviors

- 1. I am going to show you some pictures and I would like you to tell me the first thing that comes to mind when you see the picture **(Note: warm-up question. Should take ±15 min)**
- Baby being wiped after delivery
- Baby being bathed after delivery
- Skin to skin care after delivery
- Baby being breastfed immediately after delivery
- Pre-lactal feeding
- Facility delivery
- Postnatal care visit

# Theme 3: Delivery & Newborn Care

# Can you describe what role fathers in your community play in: (Note: explain them they don’t have to physically perform these tasks- but they might have a say in how it is performed by someone else)

# Deciding where delivery takes place

# Deciding how the baby is kept warm after delivery

- Deciding when the babies if first bathed
- How and when the baby is fed in the first days if life
- Whether an HEW visits in the first days of life
  1. As fathers who do you trust most to give advice about care in pregnancy and for the newborn baby?

**Theme 4: Conflicting advice and family support**

# I am going to read you a story about a mother called Aster who lives in a village like yours:

# “Aster is heavily pregnant. Aster’s family thinks that babies should be born at home, but they have been visited by an HEW who had advised them that it is safer for Aster and her baby if she delivers in the health center. Aster’s husband discusses the issue with his mother who says blood should not be shed outside of the house.

# What do you think Aster and her family will do? Probe: What do you think influenced the decision?

# Think about families like Aster’s, how are decisions made about place of delivery? Are issues discussed among the family members, or is there a main decision maker?

# Theme 5: Most significant change

- 1. What do you think have been the biggest changes in how newborns are cared for in this community in the last 2 years? What do you think influenced this change? What impact have these changes had? **(Note: focus on maternal and newborn care)**

# Theme 6: HEW and HDA

- 1. What do father in your community think about the work of the HDA and the HEW? (Probe: do they think the HEW is suitable to carry out her tasks (education, age, local knowledge)? Does she give good advice? )
  2. Do you know what information HDAs or HEWs provide to mothers regarding:
     - Where to deliver?
     - How to care for the baby immediately after they come out
     - Bathing the baby after delivery
     - Breastfeeding the baby
     - Whether to give the baby additional foods or fluids in the first days of life
  3. I am going to read out a few statements to you: As soon as you hear the statement, say the first thing that comes to your mind. You can agree or disagree with the statement, or you can comments on it. Your opinion can be different from the other participants, but there are no good or bad answers. **Do a practice round and encourage them to respond immediately.**

I am now going to read the statement:

1. **Husbands and wives should make their own decisions about newborn care, the views of others are not important**

Can you help me understand your response? Does everyone agree with the response?

1. **The HEW and HDA involve fathers in their conversations about pregnancy and care of the newborn.**

Can you help me understand your response? Does everyone agree with the response?

**Theme 7: Interviewer comments and reflections**

Include where the FGD was conducted, any interruptions, the mood during the FGD, how open the respondents were, any dominant or passive participants.

**Thank the respondents for their time**
