# Supplementary material for: Improving the use of focus group discussions in low income settings
Source: BMC Med Res Methodol. 2020 Nov 30;20:287. doi: 10.1186/s12874-020-01168-8 (PMC7706206; doi:10.1186/s12874-020-01168-8)
Supplement: Supplementary file 2 — Additional file 2. [file 12874_2020_1168_MOESM2_ESM.docx]

**Focus Group Discussion: Grandmothers**

# Theme 1: Socio-demographic and interview information

| - 1. FGD ID:   2. FGD date:   3. FGD start time:   4. FGD end time: | - 1. Interviewer code:   2. Note taker code:   3. Translator code:   4. Tape recording number: |
| --- | --- |

| **Respondent number** | **Age** | **Last completed grade** | **Occupation** | **Ethnicity & religion** | **No. of children** | **Age of youngest grandchild** |
| --- | --- | --- | --- | --- | --- | --- |
|  |  |  |  |  |  |  |
|  |  |  |  |  |  |  |
|  |  |  |  |  |  |  |
|  |  |  |  |  |  |  |
|  |  |  |  |  |  |  |
|  |  |  |  |  |  |  |

# Theme 2: Attitudes and response to the behaviors

- 1. I am going to show you some pictures and I would like you to tell me the first thing that comes to mind when you see the picture **(±15 min)**
- Baby being wrapped after delivery
- Baby being bathed after delivery
- Skin to skin care after delivery
- Baby being breastfed immediately after delivery
- Pre-lacteal feeding
- Facility delivery
- Postnatal care visit

# Theme 3: Roles and decision making

# Can you describe what role grandmothers in your village usually play in the delivery? (Probe: The baby comes out, and what happens then? Do you hold it? Do you feed it? Do you place it somewhere? What do grandmothers do?)

# I am going to show you some picture of family members from a community like yours who have welcomed a new baby to the family, the mother delivered in the house. Show pictures: This is the mother of the baby, this is the father of the baby, this is the maternal grandmother, this is the paternal grandmother. This is the HDA and this is the HEW in the community.

I would like you to work together to put the cards in a line, start with the picture of the person who had the most to the least influence on how the mother and baby are cared for. Please order the cards in relation to:

1. **When the baby is first bathed**

Help me understand your order. Do you all agree? __Would this be the same or different for a home/facility delivery? Did we miss any important influencers?

Would this order change if I ask you to rank them according to who is most influential in deciding:

1. **What is done to the baby immediately after it comes out**

Help me understand your order. Do you all agree? Would this be the same or different for a home/facility delivery? Did we miss any important influencers?

Would this order change if I ask you to rank them according to who is most influential in deciding:

1. **Feeding the baby in the first days of life**

Help me understand your order. Do you all agree? Would this be the same or different for a home/facility delivery? Did we miss any important influencers?

**Theme 4: Conflicting advice and family support**

# I am going to read you a story about a mother called Aster who lives in a village like yours:

# “Aster is heavily pregnant, Aster thinks that babies should be bathed immediately after delivery so they are clean and comfortable, but she has been visited by an HEW who had advised her that she should delay bathing the baby for at least 6 hours after delivery to help keep the baby warm”

- 1. **What do you think Aster will do?** Probe: **What do you think influenced her decision?**
  2. **Aster discusses the issue with her mother in law**

# - Says that if she does not bath the baby early people will think she is a negligent mother

# OR

# - Says that if she bathes the baby early it can get very cold and could get sick.

# What do you think Aster will do now? Probe: What do you think influenced her decision?

- 1. What type of people in your community would not behave like Aster, but behave differently? Can you explain why they behave differently?

# Theme 5: Most significant change

- 1. What do you think have been the biggest changes in how newborns are cared for in this community in the last 2 years? What do you think influenced this change? **(Note: remind them we are talking about the last 2 years, not a comparison of their daughters pregnancies and their own pregnancies )**

# Theme 6: The HEW and HDA and social networks

- 1. I am going to read out a few statements to you: As soon as you hear the statement, say the first thing that comes to your mind. You can agree or disagree with the statement, or you can comments on it. Your opinion can be different from the other participants, but there are no good or bad answers. **Do a practice round and encourage them to respond immediately.**

I am now going to read the statement:

1. **It is the responsibility of the grandmother to ensure traditional newborn care practices are adhered to.**

Can you help me understand your response? Does everyone agree with the response? Do you think most families in your community share your opinion?

1. **HDAs or HEWs know everything about how to take care of a newborn**

Can you help me understand your response? Does everyone agree with the response? Do you think most families in your community share your opinion?

1. **“Mothers of today” do not listen to your advice on how to take care of their newborns.**

Can you help me understand your response? Does everyone agree with the response? Do you think most families in your community share your opinion?

**Theme 7: Interviewer comments and reflections**

Include where the FGD was conducted, any interruptions, the mood during the FGD, how open the respondents were, any dominant or passive participants.

**Thank the respondents for their time**
