# Supplementary material for: Improving the use of focus group discussions in low income settings
Source: BMC Med Res Methodol. 2020 Nov 30;20:287. doi: 10.1186/s12874-020-01168-8 (PMC7706206; doi:10.1186/s12874-020-01168-8)
Supplement: Supplementary file 3 — Additional file 3. [file 12874_2020_1168_MOESM3_ESM.docx]

**Focus Group Discussion HEW & HDA**

# Theme 1: Socio-demographic and interview information

| - 1. FGD ID:   2. FGD date:   3. FGD start time:   4. FGD end time: | - 1. Interviewer code:   2. Note taker code:   3. Translator code:   4. Tape recording number: |
| --- | --- |

| **Respondent number** | **Age** | **Last completed grade** | **No. of years HEW/HDA** | **Ethnicity and religion** | **Marital status** | **Home Kebele** | **Home Woreda** |
| --- | --- | --- | --- | --- | --- | --- | --- |
|  |  |  |  |  |  |  |  |
|  |  |  |  |  |  |  |  |
|  |  |  |  |  |  |  |  |
|  |  |  |  |  |  |  |  |

# Theme 2: Attitudes and Norms for Behaviours

- 1. I am going to show you some pictures (Facility delivery, Immediate wrapping, immediate drying, skin-to-skin position after delivery, bathing after delivery, early breastfeeding, pre-lactal feeding, early PNC visit).

Work as a group and put the cards in two piles, one pile should be behaviors that are commonly practiced in this community and the other pile behaviors that are not. Help me understand your choices. What do you think are the reasons that the behaviors are or are not practiced?

- 1. Now I would like you to sort the cards into piles for behaviors that you think are very important for health and those that you think are less or not important. Help me understand your choices.

**Theme 3: Drivers for postnatal care**

HEWs are trained to visit women and their new babies within 3 days of delivery. We have found that this can be difficult. We would like to do an activity to identify why some families do not get visited. I will draw our answers on a tree. The canopy of the tree is the HEW visits.

- 1. What are reasons that HEW visits in the first 3 days after delivery may not happen? Any other reasons? I will put these reasons as the main roots of the trees.

**Probes:**

- Any reasons to do with HEWs knowing that someone has delivered?
- Any other reasons to do with community attitudes or not wanting to be visited?
- Any other reasons to do with logistics and time challenges?
  1. Of these reasons which are the most important? Do you all agree?
  2. Let’s work together to understand these reasons. You have said ________ is important, why do you think this reasons happens. I will put these causes on the smaller roots of the trees.
  3. Did we cover anything? Is there anything to add?

**Theme 4: Most significant change**

- 1. What do you think have been the biggest changes in how newborns are cared for in this community in the last 2 years? What do you think influenced this change?
  2. What has been the biggest change in your work in the last 2 years? How do you feel about this change?

**Theme 5: Challenges and successes of HDA/HEW work**

- 1. What are the greatest successes you have had encouraging families to change behaviors related to mothers and babies health in the last 2 years, what do you think accounts for this success?
  2. What are the greatest challenges you have faced encouraging families to change behaviors related to mothers and babies health in the last 2 years? What accounts for these challenges?
  3. How do you think the community views you and your work? What makes you say this?
  4. I am going to read out a few statements to you: As soon as you hear the statement, say the first thing that comes to your mind. You can agree or disagree with the statement, or you can comments on it. Your opinion can be different from the other participants, but there are no good or bad answers. **Do a practice round and encourage them to respond immediately.**

1. **Family members are always happy to see the HEW/HDA.**

Can you help me understand your response? Does everyone agree with the response?

1. **HEW visits do not change how babies are cared for after delivery, mothers prefer the advice of family members**

Can you help me understand your response? Does everyone agree with the response?

1. **People in the community agree with HEW/HDA advice to delay bathing the baby.**

Can you help me understand your response? Does everyone agree with the response?

# Theme 6: Interviewer comments and reflections

Include where the FGD was conducted, any interruptions, the mood during the FGD, how open the respondents were. Were all participants comfortable with each other? Was one more dominant than the other?

**Thank the respondents for their time**
