# Supplementary material for: Improving the use of focus group discussions in low income settings
Source: BMC Med Res Methodol. 2020 Nov 30;20:287. doi: 10.1186/s12874-020-01168-8 (PMC7706206; doi:10.1186/s12874-020-01168-8)
Supplement: Supplementary file 4 — Additional file 4. [file 12874_2020_1168_MOESM4_ESM.docx]

**Focus Group Discussion: Recent mothers**

# Theme 1: Socio-demographic and interview information

| - 1. FGD ID:   2. FGD date:   3. FGD start time:   4. FGD end time: | - 1. Interviewer code:   2. Note taker code   3. Translator code:   4. Tape recording number: |
| --- | --- |

| **Respondent number** | **Age** | **Last completed grade** | **Place of last delivery** | **Ethnicity and religion** | **No. of children** | **Age of youngest child** |
| --- | --- | --- | --- | --- | --- | --- |
|  |  |  |  |  |  |  |
|  |  |  |  |  |  |  |
|  |  |  |  |  |  |  |
|  |  |  |  |  |  |  |
|  |  |  |  |  |  |  |
|  |  |  |  |  |  |  |

**Theme 2: Attitudes and response to the behaviors**

- 1. I am going to show you some pictures (Facility delivery, Immediate wrapping, immediate drying, skin-to-skin position after delivery, bathing after delivery, early breastfeeding, pre-lactal feeding, early PNC visit), work as a group and put the cards in two piles, one pile should be behaviors that are commonly practiced in this community and the other pile behaviors that are not. Help me understand your choices.

- 1. Now I would like you to sort the card into piles for behaviors that you think the HDA and HEW promote and those you think that they do not promote. Help me understand your choices. For each behavior that the HEW promotes, do you think people in the community follow the advice? Tell me more about your answers? Do you all agree?

**Theme 3: Drivers for postnatal care**

HEWs are trained to visit women and their new babies within 3 days of delivery: we have found that this can be difficult.

- 1. Are most mothers visited by the HEW in the first 3 days after delivery (**Note: this is not the vaccination visit)**

*If they say not all mothers are visited:*

- 1. What could be a reason that some are not visited (Probe: might it be that they don’t know about the birth?)

**Theme 4: Conflicting advice and family support**

I am going to read you a story about a mother called Aster who lives in a village like yours: “Aster is heavily pregnant, Aster thinks that babies should be bathed immediately after delivery so they are clean and comfortable, but she has been visited by an HEW who had advised her that she should delay bathing the baby for at least 6 hours after delivery to help keep the baby warm”

- 1. What do you think Aster will do?

**Probe:** What do you think influenced her decision?

- 1. Aster discusses the issue with her mother who
- Says that if she does not bath the baby early, people will find the baby very dirty

**OR**

- Says that if she bathes the baby early it can get very cold and could get sick.

What do you think Aster will do now? **Probe:** What do you think influenced her decision?

- 1. What type of people in your community would not behave like Aster, but behave differently? Can you explain why they behave differently?

**Theme 5: Most significant change**

- 1. What do you think have been the biggest changes in how newborns are cared for in this community in the last 2 years? What do you think influenced this change?

**Theme 6: HDA and HEW**

6.1 I am going to read out a few statements to you: As soon as you hear the statement, say the first thing that comes to your mind. You can agree or disagree with the statement, or you can comments on it. Your opinion can be different from the other participants, but there are no good or bad answers. **Do a practice round and encourage them to respond immediately.**

1. **It is the responsibility of the grandmother to decide how a newborn is cared for.**

Can you help me understand your response? Does everyone agree with the response? Do you think most families in your community share your opinion?

1. **HEW visits do not change how babies are cared for after delivery, we know very well how to look after babies.**

Can you help me understand your response? Does everyone agree with the response? Do you think most families in your community share your opinion?

**Theme 7: Interviewer comments and reflections:** Include where the FGD was conducted, any interruptions, the mood during the FGD, how open the respondents were, any dominant or passive participants.

**Thank the respondent for their time**
