# Supplementary material for: Improving the use of focus group discussions in low income settings
Source: BMC Med Res Methodol. 2020 Nov 30;20:287. doi: 10.1186/s12874-020-01168-8 (PMC7706206; doi:10.1186/s12874-020-01168-8)
Supplement: Supplementary file 6 — Additional file 6. [file 12874_2020_1168_MOESM6_ESM.docx]

**Focus Group Discussion Fathers የአባቶች**

# ክፍል 1፡ ህብረተሰብዊ-ዴሞግራፊና የቃለመጠይቁ መረጃ

| - 1. የ FGD መለያ ቁጥር:   2. ቃለመጠይቅ የተደረገበት ቀን:   3. ቃለመጠይቁ የተጀመረበት ሰዓት :   4. ቃለመጠይቁ ያለቀበት ሰዓት: | - 1. የጠያቂ ኮድ:   2. ማስታወሻ መዝጋቢ ኮድ:   3. የተርጓሚ ኮድ:   4. የቀረፁ ቴፕ ቁጥር: |
| --- | --- |

| **የተጠያቂ ቁጥር** | **ዕድሜ** | **የትምህርት ደረጃ** | **ስራ** | **ብሔር እና ሃይማኖት** | **የልጅ ብዛት** | **የመጨረሻ ልጅ የተወለደበት ቦታ** | **የመጨረሻ ልጅ አድሜ** |
| --- | --- | --- | --- | --- | --- | --- | --- |
|  |  |  |  |  |  |  |  |
|  |  |  |  |  |  |  |  |

# ክፍል 2፡ ለሁኔታዎቹ ያለው አመለካከትና ምላሽ

- 1. የተወሰኑ ምስሎችን ላሳይዎ ነው እና ምስሉን ስያዩት መጀመሪያ ወደ ሃሳብዎ የሚመጣ ነገር እነዲነግሩኝ እፈልጋለው
- ከተዋለደ በኋላ እየተጠረገ ያለ ህጻን
- ከተወላ በኋላ እየታጠበ ያለ ህጻን
- ከወሊድ በኋላ የሚደረግ ቆዳ ለቆዳ እንክብካቤ
- ወድያው እንደተወለደ ጡት የሚጠባ ህጻን
- እንገር ማጥባት
- በጤና ተቋም መውለድ
- ደህረ ወሊድ ጉብኝት

# ክፍል 3፡ ወሊድ እና የጨቅላ ህጻን እንክብካቤ

# በማህበረሰባችሁ ውስጥ የአባቶች ድርሻ ምን እንደሆነ ሊነግሩኝ ይችላሉ በ-

# ወሊድ የት መከናወን እንደለበት

# ከወሊድ በኋላ ህጻኑን ከነ ሙቀቱ ማቆየት

- የህጻኑን የመጀመሪያውን ገላ መታጠብ
- ህጻኑን በህይወቱ በመጀመሪያ ቀናቶች እንዴት መጥበት እነደሚገባ
- የጤና ኤክስቴንሽን ሰራተኛ በህጻኑ የህይወቱ በመጀመሪያ ቀናቶች ጉብኝት እነድታደርግ
  1. እንደአባት፤ ለነፍሰ ጡር ና ለጨቅላ ህጻን እንክብካቤ ምክር እነዲሰጥ በይበልጥ ማንን ያምናሉ ?

**ክፍል 4፡ የሚያጋጭ ምክር እና የቤተሰብ ድጋፍ**

# ስለ አስቴር የምትባል የእርሶ አይነት መንደር ውስጥ የምትኖር እናት ታሪክ ለነብልዎ ነው :

# “አስቴር የደረሰች ነፍሰጡር ናት; የአስቴር ቤተሰቦች ህጻኑ ቤት መወለድ እነዳለበት ነው የሚያስቡት፣ግን በጤና እክስቴንሺን ሰራተኛ ተጎብኝተው አሷም ለአስቴርም ሆነ ለህጻኑ በጤና ጠቋም ብትወልድ ጤናማ እንደሆነ መክራቻዋለች፡፡ አስቴር ባል ጉዳዩን ከእናቱ ጋር ይናጋገርበታል የእናቱ አመላካከት ደግሞ ደም ከቤት ውጪ መፍሰስ የለበትም ነው፡፡

# አስቴርና ቤተሰቦቿ ምን ያደርጋሉ ብለው ያስባሉ? ያወጣጡ: ውሳኔውን ያነሳሳው ምንድን ነው ብለው ያስባሉ?

# እንደ አስቴር ያሉ ቤተሰቦችን ያስቡአቸው፣ በእናዚህ አይነት ቤተሰቦች ውስጥ የነፍሰ ጡር እና የጨቅላ ህጻን እንክብካቤ ውሳኔ እንዴት ነው የሚወሰነው? ጉዳዩን ይወያዩበታል ወይም ወና ውሳኔ ሰጪ አለ

# ክፍል 5፡ ዋናዉ ታላቅ ለውጥ

- 1. ባለፉት 2 ዓመታት ውስጥ የጨቅላ ህጻናት እንክብካቤን በተመለከተ በዚህ ማህበረሰብ ውስጥ የመጣ ትልቁ ለውጥ ምንድን ነው ብለው ያስባሉ ? ለውጡን ያነሳሳው ምንድን ነው ብለው ያስባሉ? ይህን ለውጥ ያነሳሳው ምንድን ነው ብለው ያስባሉ?

# ክፍል 6፡ የጤና ኤክስቴንሺን ሰራተኛና የጤና ልማት ሰራዊት

- 1. በዚህ መህበረሰብ ውስጥ ነፍሰጡርን፣ ወላድ ሴት እና ጨቅላ ህጻናትን በተመለከተ የጤና ልማት ሰራዊት የሚጫወቱት ዋናዎቹ ድርሻቸው ምንድን ነው? የጤና ኤክስቴንሺን ሰራተኞችስ፣ ድርሻቸው ምንድን ነው?
  2. ሰዎች ስላ ጤና ልማት ሰራዊት እና ስለ ጤና ኤክስቴንሺን ሰራተኞች ስራ ምን ያስባሉ? እንደዚህ እንዲሉ ያደረገዎ ምንድን ነው?
  3. ከ እድሜዋ፣ ትምህት ደረጃዋ፤ ስልጠናዋ፣ ልምድዋና አመለካከትዋ አንጻር ስያዩ ሰዎች የጤና ኤክስቴንሺን ሰራተኛዋ ምን ያህል ተስማሚ ናት ብለው ያስባሉ?
  4. የእርስዎ ባለቤት ቀጥለው የተዘረዘሩ ጉደዮች ከጤና ልማት ሰራዊት እና ስለ ጤና ኤክስቴንሺን ሰራተኞች መረጃ ማግኘታቸውን የውቃሉ ?
     - የት እንደሚወልዱ
     - ህጻናት ወድያው ሲወለድ እንክብካቤ እንዴት እንደሚደረግላቸው
     - ከወሊድ በኋላ የህጻኑን ገላ ማጠብ
     - ህጻኑን ጡጥ ማጥባት
     - ለህጻኑ የመጀመሪ የህይወት ቀናቶች ዉስጥ ተጨማሪ ምግቦችና ፈሳሾች መሰጠት ካለበት
  5. የተወሰኑ አረፍተ ነገሮችን ለነብልዎ ነው፡ ወድያውኑ አረፍተ ነገሩን እነደሳሙ፣ ወደ ሃሳብዎ የሚመጣውን ነገር ይናገሩ! በአረፍተ ነገሩ ሊስማሙም ላይስማሙም ይችላሉ፤ ወይን ሀሳብ ሊሰጡበት ይችላሉ፡፡ የእርሶ አመለካከት ከሌሎች ተሳታፊዎች የተለየ ሊሆን ይችላል፣ ግን መጥፎና ጥሩ መልስ የሚባል የለም፡፡ **ተራበተራ የድርጉ እናም ወድያዉኑ እንዲመልሱ ያበረታቱ!**

አረፍተ ነገሩን ለነብ ነው:

1. **ባልና ሚስት ስለ ጨቅላ ህጻን እንክብካቤ የራሳቸውን ውሳኔ መስጠር አለባቸው፤ የሌሎች አመለካከት አስፈላጊ አይደለም**

መልስዎን ልያስረዱኝ ይችላሉ? ሁሉም በዚህ መልስ ይስማማል? በእርሶ መሕበረሰብ ያሉ አባቶች ሁሉ የእርሶን ሀሳብ ይጋራሉ ብለው ያስባሉ?

1. **የጤና ኤክስቴንሽን ሰራተኛ (HEW) ና የጤና ልማት ሰራዊት (HDA) ስለ እርግዝናና የጨቅላ ህጻን አንክብካቤ ጭዉውት ውስጥ አባቶችን ያካትታሉ**

መልስዎን ልያስረዱኝ ይችላሉ? ሁሉም በዚህ መልስ ይስማማል? በእርሶ መሕበረሰብ ያሉ አባቶች ሁሉ የእርሶን ሀሳብ ይጋራሉ ብለው ያስባሉ?

1. **የጤና ኤክስቴንሽን ሰራተኞች በስራ የተወጠሩ ስለሆነች ከወሊድ በኋላ ባሉት የመጀመሪያ ቀናቶች ዉስጥ ቤተሰቡን መጥተው ለመጎብኘት አይችሉም፡፡**

መልስዎን ልያስረዱኝ ይችላሉ? ሁሉም በዚህ መልስ ይስማማል? በእርሶ መሕበረሰብ ያሉ አባቶች ሁሉ የእርሶን ሀሳብ ይጋራሉ ብለው ያስባሉ?

1. **የጤና ኤክስቴንሽን ሰራተኛ (HEW) ና የጤና ልማት ሰራዊት (HDA) ለሚስቶቻቸው የት መውለድ እነዳለባው ሲነግሩአቸው አባቶች አይወዱም፡፡**

መልስዎን ልያስረዱኝ ይችላሉ? ሁሉም በዚህ መልስ ይስማማል? በእርሶ መሕበረሰብ ያሉ አባቶች ሁሉ የእርሶን ሀሳብ ይጋራሉ ብለው ያስባሉ?

1. **የጤና ኤክስቴንሽን ሰራተኛ (HEW) ጉብኝት ከወሊድ በኋላ ያለውን የህጻን እንክብካቤ አይለውጥም፣ እኛ ህጻናትን እንዴት እንደምንካባከብ በደንብ እናዉቃለን፡፡**

መልስዎን ልያስረዱኝ ይችላሉ? ሁሉም በዚህ መልስ ይስማማል? በእርሶ መሕበረሰብያሉ አባቶች ሁሉ የእርሶን ሀሳብ ይጋራሉ ብለው ያስባሉ?

1. **በማሕበረሰቡ ያለው ህዝብ ከጤና ኤክስቴንሽን ሰራተኛ (HEW) ምክር ውስጥ ስለ የህጻናትን ገላ ማጠብ ማቆየት ላይ ይስማማሉ**

መልስዎን ልያስረዱኝ ይችላሉ? ሁሉም በዚህ መልስ ይስማማል? በእርሶ መሕበረሰብ ያሉ አባቶች ሁሉ የእርሶን ሀሳብ ይጋራሉ ብለው ያስባሉ?

**ክፍል 7፡ የጠያቂ አስተያየትና ሀሳብ**

FGD የት እነደተካሀደ ፣ማንቸውም የሚረብሹ ነገሮች፣በ FGD ጊዜ የነበረው ስሜት፣ መላሹ ምን ያህል ግልጽ እንደነበረ፣ ተናጋሪና ዝምተኛ ተሳተፊ መኖሩን ያካትታል፡፡

**መላሾቹን ስለጊዜቸው ያመስግኑቸው!**
