# Supplementary material for: Improving the use of focus group discussions in low income settings
Source: BMC Med Res Methodol. 2020 Nov 30;20:287. doi: 10.1186/s12874-020-01168-8 (PMC7706206; doi:10.1186/s12874-020-01168-8)
Supplement: Supplementary file 7 — Additional file 7. [file 12874_2020_1168_MOESM7_ESM.docx]

**Focus Group Discussion : የሴት አያቶች**

# ክፍል 1፡ ህብረተሰብዊ-ዴሞግራፊና የቃለመጠይቁ መረጃ

| - 1. የ FGD መለያ ቁጥር:   2. ቃለመጠይቅ የተደረገበት ቀን:   3. ቃለመጠይቁ የተጀመረበት ሰዓት :   4. ቃለመጠይቁ ያለቀበት ሰዓት: | - 1. የጠያቂ ኮድ:   2. የማስታወሻ መዝጋቢ ኮድ:   3. የተርጓሚ ኮድ:   4. የቀረፁ ቴፕ ቁጥር: |
| --- | --- |

| **የተጠያቂ ቁጥር** | **ዕድሜ** | **የትምህርት ደረጃ** | **ስራ** | **ብሔር እና ሃይማኖት** | **የልጅ ብዛት** | **የመጨረሻው ትንሹ የልጅ ልጅ ዕድሜ** |
| --- | --- | --- | --- | --- | --- | --- |
|  |  |  |  |  |  |  |
|  |  |  |  |  |  |  |
|  |  |  |  |  |  |  |
|  |  |  |  |  |  |  |
|  |  |  |  |  |  |  |
|  |  |  |  |  |  |  |

# ክፍል 2፡ ለሁኔታዎቹ ያለው አመለካከትና ምላሽ

- 1. የተወሰኑ ምስሎችን ላሳይዎ ነው እና ምስሉን ስያዩት መጀመሪያ ወደ ሃሳብዎ የሚመጣ ነገር እነዲነግሩኝ እፈልጋለው
- ከተዋለደ በኋላ እየተጠረገ ያለ ህጻን
- ከተወላ በኋላ እየታጠበ ያለ ህጻን
- ከወሊድ በኋላ የሚደረግ ቆዳ ለቆዳ እንክብካቤ
- ወድያው እንደተወለደ ጡት የሚጠባ ህጻን
- እንገር ማጥባት
- በጤና ተቋም መውለድ
- ደህረ ወሊድ ጉብኝት

# ከፍል 3፡ ሚናና ውሳኔ ሰጪነት

# በመንደርዎ ያሉ የሴት አያቶች ብዙ ጊዜ በወሊድ ጊዜ የሚጨወቱትን ሚና ሊያስረዱኝ ይችላሉ?

# የተወሰኑ ምስሎችን ለሳይዎ ነው አሱም እንደርሶ አይነት መህበረሰብ ውስጥ የሚኖሩ አዲስ ጨቅላ ህጻን ያገኙ ቤተሰቦች እና እናትዬዋ በቤት ውስጥ ነው የወደለደቺው፡፡ ምስሎቹን ያሳዩ: Tይቺ የህጻኑ እናት ነች፣ ይሄ የህጻኑ አባት ነው ፣ ይቺ በእናት በኩል የሴት አያት ነች፤ ይቺ በአባት በኩል የሴት አያት ነች. ይቺ የጤና ልማት ሰራተኛዋ ናት እና ይቺ ደግሞ የጤና ኤክስቴንሺን ሰረታኛዋ ነች፡፡

አሁን ከእኔ ጋር ሆነው ካርዶቹን በመስመር እንድናስቀምጥ እፈልጋለው፣ በእናትና ልጅ እንክብካቤ ላይ ትልቅ ተፅዕኖ አለው ብለው ከሚያስቡት ጀምረው ትንሽ ተፅዕኖ አለው ብለው ወደሚያስቡት ምስሎቹን ይደርድሩ፡፡ አባክዎ ካርዱን ሲደረድሩ እነዚህን ግምት ውስጥ በማስገባት ይሁን:

1. ህጻኑ ለመጀመሪያ ጊዜ ገላው ሲታጠተብ.

የእርሶን አደራደር ያስረዱኝ ፡፡ ሁላችሁም ትስማማለችሁ? ይህ በምህበረሰብዎ ላሉ ለብዙ ቤተሰቦች ይሰራል ብላው ያስባሉ? ይህ በጤና ተቋም ብሆን አንድ አይነት ወይም የተለየ የሆን ነበር? ዋና ተጽዕኖ ያለቸው ግን የረሳናቸው አሉ?

1. ከወሊድ በኋላ በህጻኑ ላይ ምን እንደሚደረግ

የእርሶን አደራደር ያስረዱኝ ፡፡ ሁላችሁም ትስማማለችሁ? ይህ በምህበረሰብዎ ላሉ ለብዙ ቤተሰቦች ይሰራል ብላው ያስባሉ? ይህ በጤና ተቋም ብሆን አንድ አይነት ወይም የተለየ የሆን ነበር? ዋና ተጽዕኖ ያለቸው ግን የረሳናቸው አሉ?

1. ህጻኑን ባሉት የህይወት መጀመሪያ ቀናቶች ውስጥ መመገብ

የእርሶን አደራደር ያስረዱኝ ፡፡ ሁላችሁም ትስማማለችሁ? ይህ በምህበረሰብዎ ላሉ ለብዙ ቤተሰቦች ይሰራል ብላው ያስባሉ? ይህ በጤና ተቋም ብሆን አንድ አይነት ወይም የተለየ የሆን ነበር? ዋና ተጽዕኖ ያለቸው ግን የረሳናቸው አሉ?

# ክፍል 4፡ ለድኅረ ወሊድ እንክብካቤ አነሳሾች

# የጤና ኤክስቴንሺን ሰረተኞች ጫቅላ ህጻናትን ከወሊድ በኋላ በሉት 3 ቀናት ውስጥ መጎብኘትን ሰልጥነዋል፡፡ ይህ ከባድ ከባድ ሊሆን እንደሚችል ሆኖ አግኝተነዋል፡፡ አንዳንድ ቤተሰቦች ለምን ጉብኝት እንደማያገኙ ለማወቅ፣ እንቅስቃሴ ማድረግ እንፈልጋልን፡፡ የዛፉ ግንድ የጤና ኤክስቴንሺን ሰረተኞች ጉብኝት ነው፡፡

# ከወሊድ በኋላ ባሉት ትንሽ ቀናቶች ዉስጥ የጤና ኤክስቴንሺን ሰረተኞች ጉብኝት የማይደረግበት ዋና ምክንያቶች ምንድን ናቸው? ሌላ ምክንያት አለ? እነዚህን ምክንያቶ እንደ ዋና የዛፉ ስር ነው የመስቀምጠቸው፡፡

# መወጣጫ:

# ማንኛዉም ምክንያት ከ አንድ ሰው መውለዱን ያወቀች የጤና ኤክስቴንሺን ሰራተኛ ጋር የተያየዘ?

# ሌላ ምክንያት ከ ምህበረሰቡ አመላካከት ጋር የተያየዘ ወይም መጎብኘት አነመፈለግ?

# ሌላ ምክንያት ከመጓጓዛ እና ጊዜ ጋር የተያየዘ?

# ከተባሉት ምክንያቶች ዉስጥ የትኞቹ አስፈላጊ ናቸው? ሁላችሁም ትስማማላችሁ?

# እንዚህን ምክንያቶች ለመረዳት አብረን እንያቸው፡፡ ______________አስፈላጊ ነው ብለው ነበር፣ ይህ ምክንያት ለምን ይፈጠራል ብለው ያስባሉ:: እነዚህን አነሳሾች ትንሹ የዛፉ ስር ላይ አስቀምጣቸዋለው.

# ሉንም ነገር ደስሰናል? የሚጨመር ማንኛውም ነገር አለ?

**ክፍል 5፡ የሚያጋጭ ምክር እና የቤተሰብ ድጋፍ**

# ስለ አስቴር የምትባል የእርሶ አይነት መንደር ውስጥ የምትኖር እናት ታሪክ ለነብልዎ ነው ፡፡

# “አስቴር የደረሰች ነፍሰጡር ናት፣አስቴር ህጻናት ንፁህና ምቹ እንዲሆኑ ወድያው ከወሊድ በኋላ የህጻኑ ገላ መታጠብ አለበት ብላ ታስባለት፣ ግን የህጸኑን ሙቀት ለማቆየት የህጻኑ ገላ መታጠብ ከወሊድ በኋለ ብያንስ ለ 6 ሰዓት መዘግየት እንዳለበት በጤና ኤክስቴንሺን ሰራተኛ ተመክራለች”

# አስቴር ምን ታደርጋለች ብለዉ ያስባሉ? ያወጠጡ: ለዚህ ዉሳኔ ያነሳሳት ምንድን ነው ብለው ያስባሉ?

#

# አስቴር ሰለዚህ ጉዳይ ከሴት አማቹዋ ጋር ተወያይታለች

# - ህፃኑኑን ቶሎ ካላጠበች ሰዎች ቸልተኛ እናት ነች የሉአታል ይላሉ

# ወይም

# - ህፃኑኑን ቶሎ ካጠበች ሊበርደውና ሊታመም ይችላል ይላሉ፡፡

# አስቴር አሁን ምን ታደርጋለች ብለዉ ያስባሉ? ያወጠጡ: ለዚህ ዉሳኔ ያነሳሳት ምንድን ነው ብለው ያስባሉ?

# በማህበረሰባችው ውስጥ ምን አይነት ሰዎች እንደ አስቴር ልሆኑ ይችላሉ? ምን አይነቶቹ ደግሞ የተለዩ ይሆናሉ?

# ክፍል 6፡ ዋናዉ ታላቅ ለውጥ

- 1. ባለፉት 2 ዓመታት ውስጥ የጨቅላ ህጻናት እንክብካቤን በተመለከተ በዚህ ማህበረሰብ ውስጥ የመጣ ትልቁ ለውጥ ምንድን ነው ብለው ያስባሉ ? ለውጡን ያነሳሳው ምንድን ነው ብለው ያስባሉ? ይህን ለውጥ ያነሳሳው ምንድን ነው ብለው ያስባሉ?

# ክፍል 7፡ የጤና ኤክስቴንሺን ሰራተኛ ፣የጤና ልማት ሰራዊትና የህብረተሰብ መገናኛዎች

- 1. የተወሰኑ አረፍተ ነገሮችን ለነብልዎ ነው፡ ወድያውኑ አረፍተ ነገሩን እነደሳሙ፣ ወደ ሃሳብዎ የሚመጣውን ነገር ይናገሩ! በአረፍተ ነገሩ ሊስማሙም ላይስማሙም ይችላሉ፤ ወይን ሀሳብ ሊሰጡበት ይችላሉ፡፡ የእርሶ አመለካከት ከሌሎች ተሳታፊዎች የተለየ ሊሆን ይችላል፣ ግን መጥፎና ጥሩ መልስ የሚባል የለም፡፡ **ተራበተራ የድርጉ እናም ወድያዉኑ እንዲመልሱ ያበረታቱ!**

አረፍተ ነገሩን ለነብ ነው:

1. **የባህላዊ የህጻናት እንክብካቤ መጠበቁን ማረጋገጥ የ ሴት አያቶች ሀላፊነት ነው፡፡**

መልስዎን ልያስረዱኝ ይችላሉ? ሁሉም በዚህ መልስ ይስማማል? በእርሶ መሕበረሰብ ያሉ ቤተሰቦች ሁሉ የእርሶን ሀሳብ ይጋራሉ ብለው ያስባሉ?

1. **ቤተሰቦች የጤና ኤክስቴንሺን ሰረተኞችና የጤና ልማት ሰራዊት የት መውለድ እነዳለባቸው ሲነግሩአቸው አይወዱም፡፡**

መልስዎን ልያስረዱኝ ይችላሉ? ሁሉም በዚህ መልስ ይስማማል? በእርሶ መሕበረሰብ ያሉ ቤተሰቦች ሁሉ የእርሶን ሀሳብ ይጋራሉ ብለው ያስባሉ?

1. **የጤና ኤክስቴንሽን ሰራተኛ (HEW) ጉብኝት ከወሊድ በኋላ ያለውን የህጻን እንክብካቤ አይለውጥም፣ እኛ ህጻናትን እንዴት እንደምንካባከብ በደንብ እናዉቃለን፡፡**

መልስዎን ልያስረዱኝ ይችላሉ? ሁሉም በዚህ መልስ ይስማማል? በእርሶ መሕበረሰብ ያሉ ቤተሰቦች ሁሉ የእርሶን ሀሳብ ይጋራሉ ብለው ያስባሉ?

1. **በማሕበረሰቡ ያለው ህዝብ ከጤና ኤክስቴንሽን ሰራተኛ (HEW) ምክር ውስጥ ስለ የህጻናትን ገላ ማጠብ ማቆየት ላይ ይስማማሉ፡፡**

መልስዎን ልያስረዱኝ ይችላሉ? ሁሉም በዚህ መልስ ይስማማል? በእርሶ መሕበረሰብ ያሉ ቤተሰቦች ሁሉ የእርሶን ሀሳብ ይጋራሉ ብለው ያስባሉ?

1. **በመህበረሱ ውስጥ ያሉ ሌሎች ሰዎች የኔን ቤተሰብ የጨቅለ እንክብካቤ ዘዴ ቢከተሉ አስፈላጊ ነው**

መልስዎን ልያስረዱኝ ይችላሉ? ሁሉም በዚህ መልስ ይስማማል? በእርሶ መሕበረሰብ ያሉ ቤተሰቦች ሁሉ የእርሶን ሀሳብ ይጋራሉ ብለው ያስባሉ?

1. **በመህበረሱ ውስጥ ያሉ ሰዎች ለሴት ለወሊድ ተመራች ቦታ የት እንደሆና ይነጋገራሉ፡፡**

መልስዎን ልያስረዱኝ ይችላሉ? ሁሉም በዚህ መልስ ይስማማል? በእርሶ መሕበረሰብ ያሉ ቤተሰቦች ሁሉ የእርሶን ሀሳብ ይጋራሉ ብለው ያስባሉ?

**ክፍል 8፡ የጠያቂ አስተያየትና ሀሳብ**

FGD የት እነደተካሀደ ፣ማንቸውም የሚረብሹ ነገሮች፣በ FGD ጊዜ የነበረው ስሜት፣ መላሹ ምን ያህል ግልጽ እንደነበረ፣ ተናጋሪና ዝምተኛ ተሳተፊ መኖሩን ያካትታል፡፡

**መላሾቹን ስለጊዜቸው ያመስግኑቸው!**
