# Supplementary material for: Improving the use of focus group discussions in low income settings
Source: BMC Med Res Methodol. 2020 Nov 30;20:287. doi: 10.1186/s12874-020-01168-8 (PMC7706206; doi:10.1186/s12874-020-01168-8)
Supplement: Supplementary file 8 — Additional file 8. [file 12874_2020_1168_MOESM8_ESM.docx]

**Focus Group Discussion : ለጤና ኤክስቴንሺን ሰረተኛና ጤና ልማት ሰራዊት**

# ክፍል 1፡ ህብረተሰብዊ-ዴሞግራፊና የቃለመጠይቁ መረጃ

| - 1. የ FGD መለያ ቁጥር:   2. ቃለመጠይቅ የተደረገበት ቀን:   3. ቃለመጠይቁ የተጀመረበት ሰዓት :   4. ቃለመጠይቁ ያለቀበት ሰዓት: | - 1. የጠያቂ ኮድ:   2. የማስታወሻ መዝጋቢ ኮድ:   3. የተርጓሚ ኮድ:   4. የቀረፁ ቴፕ ቁጥር: |
| --- | --- |

| **የመላሹ ቁጥር** | **ዕድሜ** | **የትምህርት ደረጃ** | **No. of years HEW/HDA** | **ብሄርና ሃይማኖት** | **የጋብቻ ሁኔታ** | **የቤት ቀበሌ** | **የቤት ወረዳ** |
| --- | --- | --- | --- | --- | --- | --- | --- |
|  |  |  |  |  |  |  |  |
|  |  |  |  |  |  |  |  |
|  |  |  |  |  |  |  |  |
|  |  |  |  |  |  |  |  |

ክፍል 2፡ ለሁኔታዎቹ ያለው አመለካከትና ልማዶች

- 1. የተወሰኑ ምስሎችን ላሳይዎ ነው ( በጤና ተቋም መውለድ፣ ወድያውኑ ማጥረግ፣ ወድያውኑ ማድረቅ፣ ከወሊድ በኋላ ያለ ቆዳ ለቆዳ አቀማመጥ፣ ከሊድ በኃላ ገላ ማጠብ፣ ቶሎ ጡት ማጥባት፣ እንገር ማጥባት፣ ቶሎ ድኅረ ወሊድ ክትል ማድረግ),

በቡድን ሆናችዉ ካርዶቹን ሁለት ቦታ ያስቀምጡአቸው! አነደኛው ቦታ በምህበረሰባችሁ ዉስጥ በብዛት የሚከናወን ሁኔታ አንደኛው ቦታ ደግም በብዛት የማይከናወን ይሆናል፡፡ ምርጫቹን አስረዱኝ፡፡ እነዚህ ድርጊቶች የሚፈፀሙበት የማይፈፀሙበት ዋና ምክንያት ምንድን ንዉ ብለው ያስባለ ?

- 1. choices. አሁን ደግሞ ከርዶቹን የጤና ኤክስቴንሺን ሰራተኞች ና የጤና ልማት ሰራዊት የሚያበረታቱና የማያበረታቱ ብላችሁ እንድትመድቡልኝ እፈልጋለው፡፡ ምርጫቹን አስረዱኝ

# ክፍል 3፡ ለድኅረ ወሊድ እንክብካቤ አነሳሾች

የጤና ኤክስቴንሺን ሰረተኞች ጫቅላ ህጻናትን ከወሊድ በኋላ በሉት 3 ቀናት ውስጥ መጎብኘትን ሰልጥነዋል፡፡ ይህ ከባድ ከባድ ሊሆን እንደሚችል ሆኖ አግኝተነዋል፡፡ አንዳንድ ቤተሰቦች ለምን ጉብኝት እንደማያገኙ ለማወቅ፣ እንቅስቃሴ ማድረግ እንፈልጋልን፡፡ መልሶቻችንን በዛፍ መልክ መሳል እፈልጋለው፡፡ የዛፉ ጥላ የጤና ኤክስቴንሺን ሰራተኛ ጉብኝት ይሆናል፡፡

- 1. ከወሊድ በኋላ ባሉት ትንሽ ቀናቶች ዉስጥ የጤና ኤክስቴንሺን ሰረተኞች ጉብኝት የማይደረግበት ዋና ምክንያቶች ምንድን ናቸው? ሌላ ምክንያት አለ? እነዚህን ምክንያቶ እንደ ዋና የዛፉ ስር ነው የመስቀምጠቸው፡፡

# መወጣጫ:

# ማንኛዉም ምክንያት ከ አንድ ሰው መውለዱን ያወቀች የጤና ኤክስቴንሺን ሰራተኛ ጋር የተያየዘ?

# ሌላ ምክንያት ከ ምህበረሰቡ አመላካከት ጋር የተያየዘ ወይም መጎብኘት አነመፈለግ?

# ሌላ ምክንያት ከመጓጓዛ እና ጊዜ ጋር የተያየዘ?

- 1. ከተባሉት ምክንያቶች ዉስጥ የትኞቹ አስፈላጊ ናቸው? ሁላችሁም ትስማማላችሁ?
  2. እንዚህን ምክንያቶች ለመረዳት አብረን እንያቸው፡፡ ______________አስፈላጊ ነው ብለው ነበር፣ ይህ ምክንያት ለምን ይፈጠራል ብለው ያስባሉ:: እነዚህን አነሳሾች ትንሹ የዛፉ ስር ላይ አስቀምጣቸዋለው

#

- 1. ሉንም ነገር ደስሰናል? የሚጨመር ማንኛውም ነገር አለ?

# ክፍል 4: ዋናው ታላቅ ለውጥ

- 1. ባለፉት 2 ዓመታት ውስጥ የጨቅላ ህጻናት እንክብካቤን በተመለከተ በዚህ ማህበረሰብ ውስጥ የመጣ ትልቁ ለውጥ ምንድን ነው ብለው ያስባሉ ? ለውጡን ያነሳሳው ምንድን ነው ብለው ያስባሉ? ይህን ለውጥ ያነሳሳው ምንድን ነው ብለው ያስባሉ?
  2. ባለፉት 2 ዓመታት ውስጥ በስራችሁ ትልቁ ለውጥ ምን ነበር? ስለዚህ ለውጥ ምን ይሰማችዋል?

**ክፍል 5: ለ ጤና ኤክስቴንሽን ሰረታኛ እና ጤና ልማት ሰራዊት ስራ ማነቆና ስኬት**

- 1. ባለፉት 2 ዓመታት ውስጥ ቤተሰቦች የባህሪ ለውጥ እንዲያመጡ ማበረታታት ውስጥ ፣ያገኛችሁት ትልልቆቹ ስኬቶች ምን ነበሩ፣ ለዚህ ስኬት ምክንያት የሚሆን ምንድን ነው ብለው ያስባሉ?
  2. ባለፉት 2 ዓመታት ውስጥ ቤተሰቦች የባህሪ ለውጥ እንዲያመጡ ማበረታታት ውስጥ ፣ያገጠማችሁ ትልልቆቹ ማነቆዎች ምን ነበሩ፣ ለዚህ ማነቆ ምክንያት የሚሆን ምንድን ነው ብለው ያስባሉ?
  3. ህብረተሰቡ እናንቴንና ስራችሁን እንዴት የመለከታል ብላችሁ ታስባላችሁ? እንደዚህ እንዲሉ ያደረገዎ ምንድን ነው?
  4. ወሰኑ አረፍተ ነገሮችን ለነብልዎ ነው፡ ወድያውኑ አረፍተ ነገሩን እነደሳሙ፣ ወደ ሃሳብዎ የሚመጣውን ነገር ይናገሩ! በአረፍተ ነገሩ ሊስማሙም ላይስማሙም ይችላሉ፤ ወይን ሀሳብ ሊሰጡበት ይችላሉ፡፡ የእርሶ አመለካከት ከሌሎች ተሳታፊዎች የተለየ ሊሆን ይችላል፣ ግን መጥፎና ጥሩ መልስ የሚባል የለም፡፡ **ተራበተራ የድርዱ እናም ወድያዉኑ እንዲመልሱ ያበረታቱ!**

1. **የቤተሰብ አባላት የጤና ኤክስቴንሺን ሰረተኛ/የጤና ልማት ሰራተኛ ማየት ሁሌም ያስደስታቸዋል፡፡**

መልስዎን ልያስረዱኝ ይችላሉ? ሁሉም በዚህ መልስ ይስማማል? በእርሶ መሕበረሰብ ያሉ ቤተሰቦች ሁሉ የእርሶን ሀሳብ ይጋራሉ ብለው ያስባሉ?

1. **ቤተሰቦች የጤና ኤክስቴንሺን ሰረተኞችና የጤና ልማት ሰራዊት የት መውለድ እነዳለባቸው ሲነግሩአቸው አይወዱም፡፡**

መልስዎን ልያስረዱኝ ይችላሉ? ሁሉም በዚህ መልስ ይስማማል? በእርሶ መሕበረሰብ ያሉ ቤተሰቦች ሁሉ የእርሶን ሀሳብ ይጋራሉ ብለው ያስባሉ?

1. **የጤና ኤክስቴንሽን ሰራተኛ (HEW) ጉብኝት ከወሊድ በኋላ ያለውን የህጻን እንክብካቤ አይለውጥም፣ እናቶች የቤተሰቦቻቸውን ምክር ነው የሚመርጡት**

መልስዎን ልያስረዱኝ ይችላሉ? ሁሉም በዚህ መልስ ይስማማል? በእርሶ መሕበረሰብ ያሉ ቤተሰቦች ሁሉ የእርሶን ሀሳብ ይጋራሉ ብለው ያስባሉ?

1. **በማሕበረሰቡ ያለው ህዝብ ከጤና ኤክስቴንሽን ሰራተኛ (HEW) ምክር ውስጥ ስለ የህጻናትን ገላ ማጠብ ማቆየት ላይ ይስማማሉ፡፡**

መልስዎን ልያስረዱኝ ይችላሉ? ሁሉም በዚህ መልስ ይስማማል? በእርሶ መሕበረሰብ ያሉ ቤተሰቦች ሁሉ የእርሶን ሀሳብ ይጋራሉ ብለው ያስባሉ?

**ክፍል 6፡ የጠያቂ አስተያየትና ሀሳብ**

FGD የት እነደተካሀደ ፣ማንቸውም የሚረብሹ ነገሮች፣በ FGD ጊዜ የነበረው ስሜት፣ መላሹ ምን ያህል ግልጽ እንደነበረ፣ መላሾቹ እርበርሳቸው ተመቻችተው ነበር? ተናጋሪና ዝምተኛ ተሳተፊ መኖሩን ያካትታል፡፡

**መላሾቹን ስለጊዜቸው ያመስግኑቸው!**
