# Supplementary material for: Improving the use of focus group discussions in low income settings
Source: BMC Med Res Methodol. 2020 Nov 30;20:287. doi: 10.1186/s12874-020-01168-8 (PMC7706206; doi:10.1186/s12874-020-01168-8)
Supplement: Supplementary file 9 — Additional file 9. [file 12874_2020_1168_MOESM9_ESM.docx]

**Focus Group Discussion : በቅርብ ለወለደች እናቶች**

# ክፍል 1፡ ህብረተሰብዊ-ዴሞግራፊና የቃለመጠይቁ መረጃ

| - 1. የ FGD መለያ ቁጥር:   2. ቃለመጠይቅ የተደረገበት ቀን:   3. ቃለመጠይቁ የተጀመረበት ሰዓት :   4. ቃለመጠይቁ ያለቀበት ሰዓት: | - 1. የጠያቂ ኮድ:   2. የማስታወሻ መዝጋቢ ኮድ:   3. የተርጓሚ ኮድ:   4. የቀረፁ ቴፕ ቁጥር: |
| --- | --- |

| **የተጠያቂ ቁጥር** | **ዕድሜ** | **የትምህርት ደረጃ** | **የመጨራሻ ወሊድ ቦታ** | **ብሔር እና ሃይማኖት** | **የልጅ ብዛት** | **የመጨረሻው ትንሹ የልጅ ልጅ ዕድሜ** |
| --- | --- | --- | --- | --- | --- | --- |
|  |  |  |  |  |  |  |
|  |  |  |  |  |  |  |
|  |  |  |  |  |  |  |
|  |  |  |  |  |  |  |
|  |  |  |  |  |  |  |
|  |  |  |  |  |  |  |

ክፍል 2፡ ለሁኔታዎቹ ያለው አመለካከትና ምላሽ

- 1. የተወሰኑ ምስሎችን ላሳይዎ ነው ( በጤና ተቋም መውለድ፣ ወድያውኑ ማጥረግ፣ ወድያውኑ ማድረቅ፣ ከወሊድ በኋላ ያለ ቆዳ ለቆዳ አቀማመጥ፣ ከሊድ በኃላ ገላ ማጠብ፣ ቶሎ ጡት ማጥባት፣ እንገር ማጥባት፣ ቶሎ ድኅረ ወሊድ ክትል ማድረግ), በቡድን ሆናችዉ ካርዶቹን ሁለት ቦታ ያስቀምጡአቸው! አነደኛው ቦታ በምህበረሰባችሁ ዉስጥ በብዛት የሚከናወን ሁኔታ አንደኛው ቦታ ደግም በብዛት የማይከናወን ይሆናል፡፡ ምርጫቹን አስረዱኝ
  2. አሁን ደግሞ ከርዶቹን ለጤና በጣም አስፈላጊና በጥቂት አስፈላጊ ብላችሁ እንድትመድቡልኝ እፈልጋለው፡፡ ምርጫቹን አስረዱኝ
  3. አሁን ደግሞ ከርዶቹን የጤና ኤክስቴንሺን ሰራተኞች ና የጤና ልማት ሰራዊት የሚያበረታቱና የማያበረታቱ ብላችሁ እንድትመድቡልኝ እፈልጋለው፡፡ ምርጫቹን አስረዱኝ ፡፡ የጤና ኤክስቴንሺን ሰራተኞች ለሚያበረታቱ ለማናቸውም ድርጊቶች፣ ህብረተሰቡ ምክሩን ይከተላል ብላችሁ ታስባላችወሁ? መልሶን ያብራሩልኝ? ሁላችሁም ትስማማላችሁ?
  4. ለሚከተሉት ድርጊቶች በማህበረሰብዎ ውስጥ ያሉ ሰዎች ይደግፋሉ ወይም አይደግፉም ብለው ይሚያስቡትን ይንገሩኝ፡፡

- በጤና ተቋም መውለድ

- ህጻኑን ወድያው እንደተወለደ መጥረግy

- ከወሊድ በኋላ ገላዉን ብያንስ ከ 6 ሰዓት በፊት አለማጠብ

- ከወሊድ በኋላ በሉት 3 ቀናት ዉስጥ በጤና ኤክስቴንሺን ሰራተኛ መጎብኘት

- ህጻኑ ከተወለደ በኋላ በለው 1 ሰዓት ውስጥ ጡት ማጥባት

- የሕይወቱ የመጀመሪያ 3 ቀናቶች ውስጥ ከእናት ጡት ወተት ዉጪ ምንም አይነት ምግብና ፈሳሽ አለመስጠት

- ህጻኑ 6 ወር ከመሆኑ በፊት ዉሃ መስጠት

# ክፍል 3፡ ለድኅረ ወሊድ እንክብካቤ አነሳሾች

የጤና ኤክስቴንሺን ሰረተኞች ጫቅላ ህጻናትን ከወሊድ በኋላ በሉት 3 ቀናት ውስጥ መጎብኘትን ሰልጥነዋል፡፡ ይህ ከባድ ከባድ ሊሆን እንደሚችል ሆኖ አግኝተነዋል፡፡ አንዳንድ ቤተሰቦች ለምን ጉብኝት እንደማያገኙ ለማወቅ፣ እንቅስቃሴ ማድረግ እንፈልጋልን፡፡ መልሶቻችንን በዛፍ መልክ መሳል እፈልጋለው፡፡ የዛፉ ጥላ የጤና ኤክስቴንሺን ሰራተኛ ጉብኝት ይሆናል፡፡

- 1. ከወሊድ በኋላ ባሉት ትንሽ ቀናቶች ዉስጥ የጤና ኤክስቴንሺን ሰረተኞች ጉብኝት የማይደረግበት ዋና ምክንያቶች ምንድን ናቸው? ሌላ ምክንያት አለ? እነዚህን ምክንያቶ እንደ ዋና የዛፉ ስር ነው የመስቀምጠቸው፡፡

# መወጣጫ:

# ማንኛዉም ምክንያት ከ አንድ ሰው መውለዱን ያወቀች የጤና ኤክስቴንሺን ሰራተኛ ጋር የተያየዘ?

# ሌላ ምክንያት ከ ምህበረሰቡ አመላካከት ጋር የተያየዘ ወይም መጎብኘት አነመፈለግ?

# ሌላ ምክንያት ከመጓጓዛ እና ጊዜ ጋር የተያየዘ?

- 1. ከተባሉት ምክንያቶች ዉስጥ የትኞቹ አስፈላጊ ናቸው? ሁላችሁም ትስማማላችሁ?
  2. እንዚህን ምክንያቶች ለመረዳት አብረን እንያቸው፡፡ ______________አስፈላጊ ነው ብለው ነበር፣ ይህ ምክንያት ለምን ይፈጠራል ብለው ያስባሉ:: እነዚህን አነሳሾች ትንሹ የዛፉ ስር ላይ አስቀምጣቸዋለው

#

- 1. ሉንም ነገር ደስሰናል? የሚጨመር ማንኛውም ነገር አለ?

**ክፍል 4፡ የሚያጋጭ ምክር እና የቤተሰብ ድጋፍ**

# ስለ አስቴር የምትባል የእርሶ አይነት መንደር ውስጥ የምትኖር እናት ታሪክ ለነብልዎ ነው :

አስቴር የደረሰች ነፍሰጡር ናት፣አስቴር ህጻናት ንፁህና ምቹ እንዲሆኑ ወድያው ከወሊድ በኋላ የህጻኑ ገላ መታጠብ አለበት ብላ ታስባለት፣ ግን የህጸኑን ሙቀት ለማቆየት የህጻኑ ገላ መታጠብ ከወሊድ በኋለ ብያንስ ለ 6 ሰዓት መዘግየት እንዳለበት በጤና ኤክስቴንሺን ሰራተኛ ተመክራለች”

- 1. አስቴር ምን ታደርጋለች ብለዉ ያስባሉ?

**ያወጠጡ**: ለዚህ ዉሳኔ ያነሳሳት ምንድን ነው ብለው ያስባሉ?

- 1. አስቴር ሰለዚህ ጉዳይ ከእህቶቿ ጋር ተወያይታለች፣ እነሱም

# - ህፃኑኑን ቶሎ ካላጠበች ሰዎች ቸልተኛ እናት ነች የሉአታል ይላሉ

# ወይም

# - ህፃኑኑን ቶሎ ካጠበች ሊበርደውና ሊታመም ይችላል ይላሉ፡፡

# አስቴር አሁን ምን ታደርጋለች ብለዉ ያስባሉ? ያወጠጡ: ለዚህ ዉሳኔ ያነሳሳት ምንድን ነው ብለው ያስባሉ?

- 1. በማህበረሰባችው ውስጥ ምን አይነት ሰዎች እንደ አስቴር ልሆኑ ይችላሉ? ምን አይነቶቹ ደግሞ የተለዩ ይሆናሉ?

# ክፍል 5፡ ዋናው ታላቅ ለውጥ

- 1. ባለፉት 2 ዓመታት ውስጥ የጨቅላ ህጻናት እንክብካቤን በተመለከተ በዚህ ማህበረሰብ ውስጥ የመጣ ትልቁ ለውጥ ምንድን ነው ብለው ያስባሉ ? ለውጡን ያነሳሳው ምንድን ነው ብለው ያስባሉ? ይህን ለውጥ ያነሳሳው ምንድን ነው ብለው ያስባሉ?

**ክፍል 6፡ የጤና ልማት ሰራዊትና የጤና ኤክስቴንሺን ሰራተኛ**

- 1. የተወሰኑ አረፍተ ነገሮችን ለነብልዎ ነው፡ ወድያውኑ አረፍተ ነገሩን እነደሳሙ፣ ወደ ሃሳብዎ የሚመጣውን ነገር ይናገሩ! በአረፍተ ነገሩ ሊስማሙም ላይስማሙም ይችላሉ፤ ወይን ሀሳብ ሊሰጡበት ይችላሉ፡፡ የእርሶ አመለካከት ከሌሎች ተሳታፊዎች የተለየ ሊሆን ይችላል፣ ግን መጥፎና ጥሩ መልስ የሚባል የለም፡፡ **ተራበተራ የድርጉ እናም ወድያዉኑ እንዲመልሱ ያበረታቱ!**

1. **የጨቅላ ህጻን እንክብካቤ እንዴት እንደሚደረግ ለመወሰን የሴት አያቶች ሃለፊነት ነው**

መልስዎን ልያስረዱኝ ይችላሉ? ሁሉም በዚህ መልስ ይስማማል? በእርሶ መሕበረሰብ ያሉ ቤተሰቦች ሁሉ የእርሶን ሀሳብ ይጋራሉ ብለው ያስባሉ?

1. **ቤተሰቦች የጤና ኤክስቴንሺን ሰረተኞችና የጤና ልማት ሰራዊት የት መውለድ እነዳለባቸው ሲነግሩአቸው አይወዱም፡፡**

መልስዎን ልያስረዱኝ ይችላሉ? ሁሉም በዚህ መልስ ይስማማል? በእርሶ መሕበረሰብ ያሉ ቤተሰቦች ሁሉ የእርሶን ሀሳብ ይጋራሉ ብለው ያስባሉ?

1. **የጤና ኤክስቴንሽን ሰራተኛ (HEW) ጉብኝት ከወሊድ በኋላ ያለውን የህጻን እንክብካቤ አይለውጥም፣ እኛ ህጻናትን እንዴት እንደምንካባከብ በደንብ እናዉቃለን፡፡**

መልስዎን ልያስረዱኝ ይችላሉ? ሁሉም በዚህ መልስ ይስማማል? በእርሶ መሕበረሰብ ያሉ ቤተሰቦች ሁሉ የእርሶን ሀሳብ ይጋራሉ ብለው ያስባሉ?

1. **በማሕበረሰቡ ያለው ህዝብ ከጤና ኤክስቴንሽን ሰራተኛ (HEW) ምክር ውስጥ ስለ የህጻናትን ገላ ማጠብ ማቆየት ላይ ይስማማሉ፡፡**

መልስዎን ልያስረዱኝ ይችላሉ? ሁሉም በዚህ መልስ ይስማማል? በእርሶ መሕበረሰብ ያሉ ቤተሰቦች ሁሉ የእርሶን ሀሳብ ይጋራሉ ብለው ያስባሉ?

**ክፍል 7፡ የጠያቂ አስተያየትና ሀሳብ**

FGD የት እነደተካሀደ ፣ማንቸውም የሚረብሹ ነገሮች፣በ FGD ጊዜ የነበረው ስሜት፣ መላሹ ምን ያህል ግልጽ እንደነበረ፣ ተናጋሪና ዝምተኛ ተሳተፊ መኖሩን ያካትታል፡፡

**መላሾቹን ስለጊዜቸው ያመስግኑቸው!**
