# Supplementary material for: Improving the use of focus group discussions in low income settings
Source: BMC Med Res Methodol. 2020 Nov 30;20:287. doi: 10.1186/s12874-020-01168-8 (PMC7706206; doi:10.1186/s12874-020-01168-8)
Supplement: Supplementary file 10 — Additional file 10. [file 12874_2020_1168_MOESM10_ESM.docx]

# Group 1: Natural environment

The methods in this group aim to study subjects in an environment that is minimally disturbed and feels as familiar and comfortable as possible for the research participants. This would likely make them behave more “naturally” and would increase the validity of the obtained research results.

|  | “comfortable interviews” | | “observations with minimal observer interference” | | | | “Ethnographic approach” | |
| --- | --- | --- | --- | --- | --- | --- | --- | --- |
|  | Friendship pair interviews | Casual conversations | Participant observer methods | None-participant observations | Covert Observations | Video observations | (Quasi-) ethnographic research | Visual ethnographic research |
| *Short Description* | Participants are interviewed with one or a few of their good friends/peers | Participants are very informally approached by the researcher for a "chat" rather than a sit down interview | The participants observe each other, while conducting the behaviour of interest | The trained observer is present in the research area and observes people without recruiting them | Researcher is part of the group, but researcher's status remains unknown for the group that is observed | No observer is present, but situations and behaviour is recorded with a (hidden) camera | The researcher spends a significant amount of time with the study participants to get to know them very well and identify linkages etc. that would otherwise remain hidden. | Instead of written material, the ethnographer records everything with drawings, videos, photographs etc |
| *Applicability in Frontline interaction research* | This would be a good method when interviewing young mothers, especially when they are not used to speak up, or give their opinion. They may feel more comfortable among their peers | Because of the informal setting women may be more likely to talk about their behaviour and the interaction with the community health worker (CHW). | This would be a good method to check compliance of (for example) bed net use, breastfeeding etc. | This could be use in the facilities (which women come to the facility), or at the pharmacy (buying supplements) | If a researcher from the community could be recruited, this would give an "undisturbed" image of the situation and the women's natural behaviours and interactions | Not sure this would be appropriate. It could be done in facilities or public places. | Due to the complexity of experiences, beliefs, hierarchical decision making processes etc, ethnography would be a very informative tool. However time restrictions may limit the use of this technique. | Seeing some of the interactions/behaviour could be very interesting (for example the CHW visit, a family discussion, etc) however this might be unfeasible in the study settings |
| *Main advantages* | It may overcome shyness and awkwardness | Less formal, so interviewees may be less nervous etc. | No "strangers" in the environment of the participant, so they are less likely to behave differently than normal. More "private" behaviour can be observed, which could be inappropriate otherwise | People don't know that they are studied, so will be less likely to behave differently. No time commitment asked from the women. | People don't know that they are studied, so will be less likely to behave differently. No time commitment asked from the women. | People don't know that they are studied, so will be less likely to behave differently. No time commitment asked from the women. | Unravel some hidden links that will only come out when the researcher is completely trusted by the participants. It will be a great asset in finding out more about intrinsic and extrinsic motivation for behaviour | Ditto, plus the possibility that material is interpreted by multiple researchers / experts and not just the ethnographer(s) in the area. |
| *Main disadvantages* | The women may report the same behaviour, while in fact their opinions differ | Ethical considerations.  Limited time/number of questions that can be asked. | They are not trained as observers, neither in reporting back what they have observed, so detail may be lost | Ethical considerations.  Can't observe in people's houses, or other private areas | Ethical considerations.  Women may feel a bit "betrayed" | Ethical considerations.  If the cameras are visible, this may lead to significant changes in behaviour | Very time consuming. In some settings not possible to become "part of the group" | Ditto plus ethical considerations and the fact that videoing/photography may have a significant impact on behaviour. |

# Group 2: Personal Reflexion

In this group, techniques are aiming at better reflexion of the interviewees on their own behaviour. By adequate reporting, more reflective moments and asking more “why” questions, the interviewees are encouraged to think thoroughly about why they are conducting certain behaviour. This would likely lead to more in-depth responses of the participant and a better understanding of the conscious and unconscious underlying reasons for certain behaviours.

|  | Diaries | | | Personal Questions | | In-depth asking | |
| --- | --- | --- | --- | --- | --- | --- | --- |
|  | Written diary research | Mobile phone diary | Photo Diary | Subjective Personal Introspection (SPI) | Ladder interviewing | Fishbone interviewing | Repertory Grid Technique |
| *Short Description* | The participants keep a diary of their activities, interactions and behaviour acts. | Ditto, but kept on a mobile phone | The participants capture their activities in pictures on a camera or phone | The researcher asks him/herself the question why they perform certain behaviour. | The researcher asks a "why" question. The answer is used for the next why question, etc. etc. | Questions are asked on who and what situations have influence on a certain behaviour. From there, more in-depth why questions about person's and situational influences are asked, etc. | Persons, situations, etc. that influence behaviour are listed in rows, while a scale (very important to less important is listed in the columns of a table. the participant then indicates what plays a role in their behaviour. |
| *Applicability in Frontline interaction research* | This could be applicable for pregnant women and new mothers, that describe their day, what they ate, drunk, whom they visited etc. Also details on drug taken etc could be requested. | Ditto | It could be used for less personal activities/behaviours such as visiting the facility, visiting the pharmacy. Attending a health promotion session, meeting the CHW etc. | Women could be asked to think about their behaviour while acting (giving breastfeeding, attending the clinic, taking iron supplements etc) to trigger their cognitive capacities | This could be used in the situation that data collectors yield very shallow answers in normal interviews and don't get to the deeper layers of stimuli for certain behaviour | Similar to ladder interviewing - also links between different household members etc could be studied, decision making processes, etc. | Similar to fishbone and ladder techniques, however, relative importance of situations and people is measured as well, as one person is rated against another |
| *Main advantages* | Participants can keep a very detailed logbook of what they have been doing, how they felt, etc. | Could be easier to apply than the written one: they can report directly after an event and don't have to wait till they come home . If speech input is an option on the phone, this open up the participation of illiterate participants | Not restricted to literate population. It may be easier to address a sensitive issue, as they don't have to describe / talk about it in front of the interviewer but just capture it in the picture. | It is partly "ethnographic", cause the researchers know their own lives very well, but may not have thought about certain linkages, intrinsic stimuli etc. When interviewing after the SPI process probably more in-depth understanding could be obtained | Participant s are forced to think about their own behaviour in more depth. It will lead to a better understanding of behaviour. | It is a very structured way of interviewing which makes sure not to miss out on any detail, link, interaction etc that leads to certain behaviour. | Ditto |
| *Main disadvantages* | Illiteracy, lack of steering, so the diaries could contain a lot of less useable information. Lack of awareness of what is important to report | Not everyone has a phone / smart phone. They may be quicker in writing than typing messages. | Less details on feelings, emotions, etc in the pictures. It would involve others to take the pictures, hence could influence behaviour | It does require a basic level of cognitive capacity, which may sometimes be difficult in areas with limited education and/or cognitive stimulation. | The repeated "why" question may be annoying or embarrassing for the participant. | The in-depth questions may be experienced as invasive or embarrassing | Ditto |

# Group 3a: Triggering by showing - normal examples

With methods listed in this group researchers aim to “provoke” gut reactions of participants, minimising the chance of giving socially desired answers may not fully represent the truth. Participants indicate, based on examples, what is important to them, on an absolute or a relative scale. To trigger responses, images, words, narratives etc. are shown/performed to the participants, on which they are asked to comment. Furthermore, a “third person” technique could be used, to offer participants the opportunity to comment on “the woman in the play” or “the boy in the picture” etc. This could make the exercise less personal and could reduce the urge to defend oneself while talking about sensitive topics. An associative element could be added to help identify intrinsic and extrinsic motivation for conducting or avoiding certain behaviours.

|  | Performative | | Imagery | | | Words/statements | | | |
| --- | --- | --- | --- | --- | --- | --- | --- | --- | --- |
|  | Drama Performance (complete and incomplete) | Prediction Methodology | Associative imagery | Speech balloons | Participatory ranking | Associative words/grids | Incomplete story telling | Q-set statements | Interpretive description (“scenario testing”) |
| *Short Description* | A certain situation with the behaviour in it is performed in a play. Participants are asked to react on the play, or (if incomplete) to play the end of it themselves. | The participants are given a scenario (e.g. a birth) and assets (tools, money, time etc.) and will be asked what they would do in a certain situation with the given assets. | Participants are shown images and asked to respond with the first thing that comes to their mind when seeing the image | Participants are shown a situation/picture where people have empty speech balloons. They are then asked to fill in what they think the person says/thinks | Words, behaviours, acts etc are written down on (e.g.) cardboard or drawn in pictures. Participants (groups or individuals) are then asked to rank them according to importance/preference | Participants are read out some words and asked to respond with the first thing that comes to their mind when hearing a word. In the grid method, multiple associative words can be mentioned. | A story is told to the participants which they have to complete | A set of statements about a certain maternal or neonatal issue is read out to the participants and they are asked to comment on it | This is similar to the Q-set method, however the researcher describes a whole scenario, which may or may not apply to the (group of) interviewees. |
| *Applicability in frontline interaction research* | This could be a really good tool to measure mothers understanding and behaviour in case of complications, certain controversial behaviours etc. | This could be used in studying when and why people would choose for facility based care, as well as the uptake of some drugs that they need to purchase. | This could be used to "test" the reaction on good and bad practices. Or when several things happen in a picture, what is picked up first. | This could help in taking about more sensitive topics, such as getting an HIV test. | This would give a great overview of priority setting in MCH, as women have to compare activities and give them a relative score | This would be useful when exploring what is on the women's mind regarding newborn health and healthy behaviour during pregnancy | This would be useful to gather insight using "what if-scenarios". What would the woman do if she would be in the situation created by the story teller | This method could be used to check opinions of less practiced behaviours (in positive or negative sense) | This could be used when the researchers already have quite a clear picture of what is happening and "check" until there are no further changes proposed by the respondents. |
| *Main advantages* | In contrast with interviewing the players in the drama are the main characters. After it is easier to reflect on what "she" needs to do than reflecting on own (unhealthy) behaviour. | It would show directly what people find important, because they have limited resources. | It tests a gut reaction, which is most probably less prone to social desirability | People can comment in the 3rd rather than the 1st person, which makes it easier to talk about sensitive topics | All aspects relevant to the study are discussed as they are part of the ranking items. | It tests a gut reaction, which is most probably less prone to social desirability. In the grid method there is no restriction to only one associative concept/behaviour | See drama performance | People are triggered to reply and take a side (against/in favour). It may be easier to comment on a statement than formulate a original opinion about a certain topic. | Similar to Q-set method. |
| *Main disadvantages* | It will give a less clear picture on what the women practice themselves, although it does show their knowledge on certain topics | Since it is a simulated situation is gives a less clear overview of what women decide in their own situation. | It tests the most important response, but may miss out on other associations that people have in second instance | There is limited space to measure all thought they have, they have to select their most prominent one. | There is no original input from the women themselves anymore, so difficult to test if | Since people are restricted to single words and concepts no motivational links can be identified | See drama performance | Topics are probed, so less of a trigger to the women to come up with new original statements/opinions | Scenario is probed, so less of a trigger to the women to come up with new original statements/opinions |

**Group 3b: Triggering by showing – “extreme” examples & experiences**

In this group methods aim to trigger responses by showing “extreme” examples, or sharing important/significant experiences. To work with extremes (best, worst, most, least, critical) participants will be forced to “rank” certain issues in their mind and list the ones that are more/most important to them.

|  | “autobiographic examples” | | | “opinions of others” | |
| --- | --- | --- | --- | --- | --- |
|  | Critical Incident | Most Significant Change (MSC) | Most significant Pictures (ZMET) | Positive Deviance | Triangular group interviews |
| Short Description | Participants are asked to tell the researcher about a critical incident they may have experience (e.g. death of a child, sepsis, miscarriage etc.) This could also be an example from someone else. Then the respondents are asked why this has happened or what their opinion about the incident is. | Participants are asked about what they think is the most important change in their lives since intervention X. | Similar to MSC, however participants are asked to take picture of what is important to them in terms of Maternal and Newborn Health(MNH) | The behaviour of one or a few individuals that conduct a certain behaviour / activity that is different from the majority of the society they live in, but has positive effect on their health / the neonate's health. | Interviews are held with 3 participants with strong (and opposite) opinions. Because the group is small, al opinions will be heard, and domination of the discussion by an(few) individual(s) is not likely to happen when only 3 people are present. |
| Applicability in Frontline interaction research | This could be used to study preventative healthy behaviour during pregnancy and delivery and the knowledge of women around complications | This could be used for studying impact of the intervention, as well as insight in why women choose to perform the behaviour | It would be useful in terms of significant impact in the home, or other private places. | This could be used for more sensitive topics, such as HIV testing, STI care, etc. | This tool would be useful in unravelling why people act in a certain way. Because of the small group and the differences in opinions, people are forced to further explain themselves |
| Main advantages | It is something that is perceived as critical, and may therefore yield high response | More insight what is perceived as important | It might be easier to take a picture of something that is sensitive to talk about than to discuss it in words with the interviewer. It gives the participant some more time to think about what is important to them. | Issues are addressed in 3rd person, rather than 1st, which makes it easier to comment on it. It is known that the unique behaviour had its positive impacts, so people are triggered to think about their motivation not to do so. | People are forced to give arguments for their own behaviour which unravels their stream of thought a bit more. |
| Main disadvantages | It may be a sensitive topic to talk about for the women. The session may get very emotional | Only addresses one issue per participant and loses out on details on other important impacts. | There is less steering during data collection, so picture may not be representative of what is really important to the interviewees. People may change their behaviour because of the camera. | Ethical issues, it may cause further polarisation of opinions | Participants have to be selected carefully as social hierarchy may make it difficult for people to speak out in such a small group |

# Group 4: Open mind

This group of methods focuses on unbiased data collection on the targeted behaviour. The researcher does not make any assumptions and interferes as least as possible with what the participant wants to say. This minimises a researcher bias, and opens the opportunity for the participants to mention those issues that are important to them.

|  | “unbiased interviewing” | | | |
| --- | --- | --- | --- | --- |
|  | Narrative interviewing | Grounded theory methodology | Phenomenological interviewing | Theme exploration |
| Short Description | Participants are asked to tell the whole story of a certain event or time period, for example their pregnancy. | There is no research question or hypothesis. People are just asked what they think is important in their area regarding MNH | Participants are asked how they experience a certain phenomenon. | Participants are asked about their opinions or stories in certain major research themes |
| Applicability in Frontline interaction research | This could be used to explore common practices and habits during pregnancy, delivery and newborn care. | This would be a good first step to identify themes, in case only little is known about uptake of a certain intervention. |  | This would be a good first step explore certain main themes, in case only little is known about uptake of certain interventions. |
| Main advantages | It is unbiased, there are no questions that can push the respondents into a certain | Free of researcher bias | It gives more insight in the feelings and emotions of a participant, rather than the facts of behaviour or decision making | Great way of identifying initial themes |
| Main disadvantages | People may go in unnecessary details, or leave out what seems completely obvious to them , but it has been important for the research, | Lack of theory and question may make the conversations very unstructured and important issues may be missed | People in certain settings may not be used to the way of interviewing in this way and may not have the cognitive capabilities to express themselves properly. | The open character may cause loss of detail and explanations/motivations |

# Group 5: Other

These methods, could not be listed under the above five (sub) categories, but also try to obtain more unbiased in-depth information on intrinsic and extrinsic motivation for behaviour from the participants.

|  | “special target groups” | “using all expertise” | “group brainstorm” |
| --- | --- | --- | --- |
|  | Elite interviewing | Serial Moderating Interviews | Tremor Panel Research |
| Short Description | The hierarchical elite is interviewed about certain MCH issues and behaviours | In group discussion the moderator is changed per topic, to have a real expert leading each section of the discussion/focus group | Participant together address an MCH problem and jointly come up with the best (context-specific) solution to the problem |
| Applicability in Frontline interaction research | If there is a strict hierarchical structure, it would be interesting to find out what the elite 's opinion are about a certain topic. Although other women may not always say so this could have a significant impact on the choices other women make regarding MNH behaviour. |  |  |
| Main advantages | This method will obtain opinions and visions of the most influential people in a certain society | An expert would get the maximum out of the focus group and will know what questions to ask | Very practical |
| Main disadvantages | They are not representative for all women; often have more resources to spend, and could therefore act quite different from other women | It does not help the interviewer participant relationship/trust, might be confusing and is difficult to organise from an HR point of view | Not very useful for a data collection phase, more implementation of new interventions |

# Explanation Grouping

Collecting high quality and valid data on sensitive topics, such as neonatal health, is often complicated by the participants’ emotional feelings, such as pride, happiness, guilt, shame, embarrassment, shyness, etc. Furthermore, people often feel the social pressure of giving the “correct” answers. There are a few common threats for data collection on sensitive topics that researchers should try to avoid with creative data collection techniques. In the field of maternal and neonatal health (in resource poor settings) these threats could include:

1. **Social desirability** – It is often theorized that in general all mothers want to be “good mothers” and want to be recognised as a good mother by other people in their social network. If breastfeeding is well accepted in the social network and seen as something healthy for your baby, mothers will not be very keen on reporting their decision to bottle feed the child. Questions could be skipped or participation could be refused. In some cases the mother will prevaricate when a sensitive question is asked to make sure she comes across as a good mother.
2. **Reporting error –** Reporting errors could have various underlying reasons. The mother may not remember a certain behaviour or practice, she may not know the answer to a certain question about her behaviour (but still feel pressured to answer something), or she may alter her answers due to social desirability pressure (as discussed above). The likelihood that reporting error occurs, and its magnitude depend on a number of factors, such as:
   1. **Environment in which the data collection takes place –** which could again be split up into:
      - ***The physical environment of the interview/group discussion –*** *Is the interviewee familiar with the environment, are people watching? Is it appropriate for her to be there?*
      - ***Presence of peers / people they trust –*** *Who else is in the interview/group discussion? What is the relationship between the people present?*
      - ***Comfortable relationship with interviewer –*** *What is the sex of the interviewer? Does the participant feel comfortable with the interviewer, regarding his sex, ethnicity, age, socio-economic status, etc.? Self-reporting may be a more comfortable way of reporting for some women.*
      - ***Time of the day -*** *Is it appropriate for her to be there at that time? Can she still go home? Is she tired? Is she supposed to perform other tasks (at home) at that time? Etc.*
   2. **Recall bias** – *How long ago was the behaviour of interest performed? Is it unusual behaviour, or a habit? Is it likely that she remembers habits from a while ago? What methods could be used to refresh her memory?*
   3. **The topic about which questions are asked –** which could again be split up into categories such as:
      - ***"Shameful topics" –*** *Could the questions cause embarrassment for the mothers? Could it cause shyness due to the presence of other women in the interview/group discussion*
      - ***Illegal behaviour –*** *Is the behaviour against certain rules, regulations or laws? Could it result in punishments, fines, etc. if certain people find out about certain behaviour of the interviewee?*
      - ***Difficult to report –*** *Is the behaviour a habit and is it difficult to report on frequency etc. of this behaviour? Is the behaviour so irregular that it is difficult to report on?*
3. **Limited depth in Answers (Limited cognitive capabilities) –** Women in resource poor settings, with limited educational opportunities, may not often be challenged from a cognitive point of view. It may be difficult for them to reflect on their own behaviour, and give in-depth arguments and reasoning for certain behaviour. Therefore it may be difficult for the researchers to identify the true underlying factors of certain behavioural patterns. Adapted data collection / interviewing techniques may partly overcome these problems.
4. **Non-reporting bias –** Due to several reasons certain aspects of MNH behaviour may remain underexposed in the research because it has not come up in the discussions, interviews etc. held with the study participants. This could be the result of the personal character of the interviewee (is she likely to speak up, is she shy, etc.?), but could also be related to the topic discussed and the (lack of) awareness that certain information is important or someone prefers not to talk about it.
   1. **Character, socio-economic status etc. of the interviewee –** Hierarchical differences may make it difficult for some women speak up, even when feeling comfortable with the interviewer. They may not be used to be asked for their opinion, and may find it difficult to comment on especially more sensitive topics.
   2. **Topic discussed –** Which could be split up into:
      - ***Sensitive –*** *If the “wrong” behaviour is performed, women may not answer certain questions, which would lead to a non-reporting bias.*
      - ***Not aware of importance –*** *Women may not be aware that certain information (that they perceive as completely logic or normal) is very important input for the study. Probing techniques, with words, images, or performances may trigger them to comment on situations and behaviours that would not have come up directly in interviews, as it was not perceived as something worth saying by these women.*

**Researcher bias –** As is it difficult to blind researchers in most community based MNH research, researcher bias may play a role. Especially when probing techniques, etc are used, the researchers may push the participant too much to a certain direction, or may interpret statements in a subjective way. This could also partly be overcome when using the appropriate data collection methods.

| **Group** | **Theme** | **Mechanism/Technique** | **Threats decreased/minimised per data collection technique** | | | | | | | | | |
| --- | --- | --- | --- | --- | --- | --- | --- | --- | --- | --- | --- | --- |
|  |  |  | **Social Desirability** | **Uncomfortable area** | **Uncomfortable with other participants** | **Uncomfortable with interviewer** | **Recall bias** | **Lack of response due to embarrassment** | **Lack of response due to unawareness** | **Answers of limited depth** | **Non-reporting bias** | **Researcher bias** |
| Group 1 | Comfortable interviews | Friendship pair interviews |  | x | x |  |  | x |  |  |  |  |
|  |  | Casual conversations | X | x |  | x |  |  |  |  |  |  |
|  | Observations with minimal observer interference | Participant observer methods | X | x | x | x |  | x |  |  | x |  |
|  |  | None-participant observations | X | x | x | x |  | x |  |  | x |  |
|  |  | Covert Observations |  |  |  |  |  |  |  |  |  |  |
|  |  | Video observations | X | x | x | x |  | x |  |  | x | x |
|  | Ethnographic approach | Quasi-ethnographic research |  |  |  |  |  |  |  |  |  |  |
|  |  | Visual ethnographic research |  |  |  |  |  |  |  |  |  |  |
| Group 2 | Diaries | Written diary research |  |  |  |  | x |  | x | x |  | x |
|  |  | Mobile phone diary |  |  |  |  | x |  | x | x |  | x |
|  |  | Photo Diary | X |  |  |  | x |  | x | x |  | X |
|  | Personal questions | Subjective Personal Introspection |  |  |  | x |  |  |  | x | x |  |
|  |  | Ladder interviewing |  |  |  |  | x |  | x | x |  |  |
|  | In-depth interviews | Fishbone interviewing |  |  |  |  | x |  | x | X |  |  |
|  |  | Repertory Grid Technique |  |  |  |  |  |  | x | x |  |  |
| Group 3A | Performative | Drama Performance <(in)complete> |  | x |  |  |  | X |  | x | x | X |
|  |  | Prediction Methodology |  |  |  |  |  |  | x | x | x |  |
|  | Imagery | Associative imagery | X |  |  |  |  |  | x |  |  |  |
|  |  | Speech balloons (3rd person technique) |  |  |  |  |  | x |  |  |  | x |
|  |  | Participatory ranking |  |  |  |  | X |  | x | x | x | X |
|  | Words/statements | Associative words/grids | X |  |  |  |  | x |  | x | x |  |
|  |  | Incomplete story telling | X | x |  |  |  | x |  | x | x |  |
|  |  | Q-set statements | X |  |  |  | x |  | x | x |  |  |
|  |  | Interpretive description (“scenario testing”) |  |  |  |  |  |  | x |  |  |  |
| Group 3B | Autobiographic examples | Critical Incident | X |  |  |  | X |  | x | X |  |  |
|  |  | Most Significant Change | X |  |  |  | X |  |  |  |  |  |
|  |  | Most significant Pictures (ZMET) | X | x |  | x | x |  | x |  | x | X |
|  | Opinion of others | Positive Deviance | X |  |  |  | x | x |  |  |  |  |
|  |  | Triangular group interviews | X |  |  |  |  | x | x | x | x | X |
| Group 4 | Unbiased interviewing | Narrative interviewing |  |  |  |  |  |  |  | x |  | X |
|  |  | Grounded theory methodology |  |  |  |  |  |  |  | x |  | X |
|  |  | Phenomenological interviewing |  |  |  |  |  |  |  | x |  | X |
|  |  | Theme exploration |  |  |  |  |  |  |  | X |  | x |
| Group 5 | Special target groups | Elite interviewing | X |  | x | x |  | x |  |  |  |  |
|  | Using all expertise | Serial moderating interviews |  |  |  | x |  |  |  | X |  |  |
|  | Group brainstorm | Tremor Panel Research |  |  | x |  |  |  |  |  |  |  |

## Data collection Method – Threat Matrix

## Data collection Method – Target Behaviour Matrix

| **Group** | **Theme** | **Mechanism/Technique** | **Target Behaviour** | | | | | | | | | | | | |
| --- | --- | --- | --- | --- | --- | --- | --- | --- | --- | --- | --- | --- | --- | --- | --- |
|  |  |  | *Pregnant Woman* | | | | | | *Delivery* | | *Neonatal Care* | | | | |
|  |  |  | Early antenatal car seeking | HIV-test / STI test | Tetanus vaccination | Iron supplementation | Malaria prevention | Facility delivery | Prepare /buy kit for clean delivery | Facility care in case of complications | Immediate and exclusive breastfeeding | Recognition of danger signs | Delayed bathing | Malaria prevention | Skin-to-skin care |
| Group 1:  Ensuring natural environment | Comfortable interviews | Friendship pair interviews |  |  |  |  |  |  |  |  |  |  |  |  |  |
|  |  | Casual conversations |  |  |  |  |  |  |  |  |  |  |  |  |  |
|  | Observations with minimal observer interference | Participant observer methods |  |  |  |  |  |  |  |  |  |  |  |  |  |
|  |  | None-participant observations |  |  |  |  |  |  |  |  |  |  |  |  |  |
|  |  | Covert Observations* |  |  |  |  |  |  |  |  |  |  |  |  |  |
|  |  | Video observations (at facility) |  |  |  |  |  |  |  |  |  |  |  |  |  |
|  | Ethnographic approach | Quasi-ethnographic research |  |  |  |  |  |  |  |  |  |  |  |  |  |
|  |  | Visual ethnographic research |  |  |  |  |  |  |  |  |  |  |  |  |  |
| Group 2: Stimulating personal reflexion /cognitive thinking | Diaries | Written diary research |  |  |  |  |  |  |  |  |  |  |  |  |  |
|  |  | Mobile phone diary |  |  |  |  |  |  |  |  |  |  |  |  |  |
|  |  | Photo Diary |  |  |  |  |  |  |  |  |  |  |  |  |  |
|  | Personal questions | Subjective Personal Introspection** |  |  |  |  |  |  |  |  |  |  |  |  |  |
|  |  | Ladder interviewing |  |  |  |  |  |  |  |  |  |  |  |  |  |
|  | In-depth interviews | Fishbone interviewing |  |  |  |  |  |  |  |  |  |  |  |  |  |
|  |  | Repertory Grid Technique |  |  |  |  |  |  |  |  |  |  |  |  |  |
| Group 3A:  Triggering reactions (day-to-day examples) | Performative techniques | Drama Performance <(in)complete> |  |  |  |  |  |  |  |  |  |  |  |  |  |
|  |  | Prediction Methodology |  |  |  |  |  |  |  |  |  |  |  |  |  |
|  | Imagery | Associative imagery |  |  |  |  |  |  |  |  |  |  |  |  |  |
|  |  | Speech balloons |  |  |  |  |  |  |  |  |  |  |  |  |  |
|  |  | Participatory ranking |  |  |  |  |  |  |  |  |  |  |  |  |  |
|  | Words/statements | Associative words/grids |  |  |  |  |  |  |  |  |  |  |  |  |  |
|  |  | Incomplete story telling |  |  |  |  |  |  |  |  |  |  |  |  |  |
|  |  | Q-set statements |  |  |  |  |  |  |  |  |  |  |  |  |  |
|  |  | Interpretive description (“scenario testing”) |  |  |  |  |  |  |  |  |  |  |  |  |  |
| Group 3B: Triggering reactions (extremes) | Autobiographic examples | Critical Incident |  |  |  |  |  |  |  |  |  |  |  |  |  |
|  |  | Most Significant Change |  |  |  |  |  |  |  |  |  |  |  |  |  |
|  |  | Most significant Pictures (ZMET) |  |  |  |  |  |  |  |  |  |  |  |  |  |
|  | Opinion of others | Positive Deviance |  |  |  |  |  |  |  |  |  |  |  |  |  |
|  |  | Triangular group interviews |  |  |  |  |  |  |  |  |  |  |  |  |  |
| Group 4: unbiased interview | Unbiased interviewing | Narrative interviewing*** |  |  |  |  |  |  |  |  |  |  |  |  |  |
|  |  | Grounded theory methodology*** |  |  |  |  |  |  |  |  |  |  |  |  |  |
|  |  | Phenomenological interviewing*** |  |  |  |  |  |  |  |  |  |  |  |  |  |
|  |  | Theme exploration*** |  |  |  |  |  |  |  |  |  |  |  |  |  |
| Group 5: Other | Special target groups | Elite interviewing |  |  |  |  |  |  |  |  |  |  |  |  |  |
|  | Using all expertise | Serial moderating interviews**** |  |  |  |  |  |  |  |  |  |  |  |  |  |
|  | Group brainstorm | Tremor Panel Research |  |  |  |  |  |  |  |  |  |  |  |  |  |

Green: High potential for overcoming social desirability bias, Orange: Medium potential Red: Low potential; Grey: not scored as unlikely method for study *Logistically unlikely **Very unlikely for this study (e.g. pregnant researchers); *** Depending on context and what is appropriate; **** Might not be feasible from staff/logistic point of view.
